# Transcriptomic changes with increasing algal symbiont reveal the detailed process underlying establishment of coral-algal symbiosis

**DOI:** 10.1038/s41598-018-34575-5

**Published:** 2018-11-14

**Authors:** Ikuko Yuyama, Masakazu Ishikawa, Masafumi Nozawa, Masa-aki Yoshida, Kazuho Ikeo

**Affiliations:** 1 Faculty of Life and Environmental Sciences, University of Tsukuba, 111 Tennodai, Tsukuba, Ibaraki, 305–8577 Japan; 2 Department of Medical Biochemistry and Biophysics, Karolinska Institutet, Solna, 17 177 Sweden; 3 Department of Biological Sciences, Graduate School of Science, Tokyo Metropolitan University, 1–1 Minamiosawa, Hachioji, Tokyo, 192–0397 Japan; 4 Marine Biological Science Section, Education and Research Center for Biological Resources, Faculty of Life and Environmental Science, Shimane University, 194 Kamo, Okinoshima-cho, Oki, Shimane, 685-0024 Japan; 5 Center for Information Biology, National Institute of Genetics, 1111 Yata, Mishima, Shizuoka, 411–8540 Japan; 6 Center for Genomics and Bioinformatics, Tokyo Metropolitan University, 1-1 Minamiosawa, Hachioji, Tokyo, 192-0397 Japan

## Abstract

To clarify the establishment process of coral-algal symbiotic relationships, coral transcriptome changes during increasing algal symbiont densities were examined in juvenile corals following inoculation with the algae *Symbiodinium goreaui* (clade C) and *S. trenchii* (clade D), and comparison of their transcriptomes with aposymbiotic corals by RNA-sequencing. Since *Symbiodinium* clades C and D showed very different rates of density increase, comparisons were made of early onsets of both symbionts, revealing that the host behaved differently for each. RNA-sequencing showed that the number of differentially-expressed genes in corals colonized by clade D increased ca. two-fold from 10 to 20 days, whereas corals with clade C showed unremarkable changes consistent with a slow rate of density increase. The data revealed dynamic metabolic changes in symbiotic corals. In addition, the endocytosis pathway was also upregulated, while lysosomal digestive enzymes and the immune system tended to be downregulated as the density of clade D algae increased. The present dataset provides an enormous number of candidate symbiosis-related molecules that exhibit the detailed process by which coral-algal endosymbiosis is established.

## Introduction

The association between scleractinian corals and algal symbionts (*Symbiodinium* spp.), is essential to primary production and reef building in tropical and subtropical oceans. This symbiotic relationship has attracted significant attention due to its sensitivity to environmental changes, often breaking down due to environmental stressors, such as high temperature, high CO_2_ levels or pollution^1–4^. The breakdown of coral-algal endosymbiosis leads to the “coral bleaching” phenomenon, whereby corals appear colorless and damage may ultimately be fatal^1,2,5^. Corals and the endosymbiont dinoflagellate *Symbiodinium* live in a mutualistic relationship, corals receiving amino acids, fatty acids and photosynthetic products, such as glucose, from the symbiont, which in turn effectively uses nitrogen and phosphorus from host cells^6–16^. In addition, *Symbiodinium* are protected against harmful UV radiation by ultraviolet-absorbing substances, such as mycosporine-like amino acids (MAAs), in the corals^17–19^. Although the details of coral–algal endosymbiotic relationships are becoming better known, the mechanisms of endosymbiont establishment are still remain the questions.

Recently, information on the mechanisms involved in the symbiosis of a cnidarian and *Symbiodinium* is gradually accumulating^20,21^. During the initiation of endosymbiosis, coral lectin binds specific glycans on the algal cell wall, the lectin/glycan interaction being a key process for the acquisition of specific algae^22–24^. Acquired algae in corals are surrounded by membrane, a so-called symbiosome, on which Rab family proteins enable the persistence of healthy algal cells and exclusion of dysfunctional algae^25–27^. In addition, previous studies have shown that the TGFβ signaling pathway and Tsr proteins are also involved in the maintenance of algae in cnidarian tissue^28,29^. Moreover, genes whose expression is up or downregulated between aposymbiotic and symbiotic stages have been identified as candidate symbiosis-related molecules. Genes encoding transporters, carbonic anhydrase, cell membrane proteins, metabolism-associated proteins and peroxidase, plus those related to apoptosis and the inflammatory response, have been identified as endosymbiosis-related genes^30–35^. Although studies involving transcriptomic analyses have become more common over the past decade, the detection of symbiosis-related genes is difficult, some reports showing little difference between symbiotic and non-symbiotic states^31,32^. Previous studies focused on the very early symbiotic state of planura larvae^31^ or on the corals colonized with symbiont at high density^34,35^, but few studies have investigated the process of increasing algal density in the corals. Because the latter process may involves key mechanisms for establishing endosymbiosis being expressed inside corals, it is possible that the molecular process underlying such will be further clarified by studying the genes whose expression changes with increasing *Symbiodinium* density.

In a previous study, we experimentally prepared corals (*Acropora tenuis*) associated with monoclonal *Symbiodinium* [*S. goreaui* (clade C1) or *S. trenchii* (clade D1a)] in the laboratory^36^. Their closely related genotype, clade C1, C2, and D, are naturally associated with *A. tenuis* in the field, and clade D has been characterized as stress tolerant^37–39^. In this trial, clade C symbionts hardly increased in corals during the first 2 months after inoculation, whereas clade D symbionts increased quickly^36^, implying that the process of symbiont establishment differs considerably depending on the genetic type (clade) of *Symbiodinium*. A comparison of these two symbiotic systems therefore provides a clue to understanding the complicated mechanisms underlying endosymbiosis establishment. Next generation sequencing provides larger scale transcriptomic information about both corals and *Symbiodinium*. In addition to large transcriptomic datasets, whole coral and algal genome sequences have also become available^40–46^, which are helpful for distinguishing the transcriptomes of host corals and symbionts from the coral–algae complex. Here, we generated RNA-seq data using the scleractinian coral *Acropora tenuis* in the early endosymbiosis stage (approximately 10 days and 20 days after the start of co-incubation following inoculation) with clade C and clade D *Symbiodinium*. We investigated the algal symbiont-dependent changes in gene expression by comparing transcriptomes between the aposymbiotic and symbiotic to clarify the establishment of symbiosis.

## Results and Discussion

### Algal cells were broken during the early symbiosis stage

**Figure 1:**
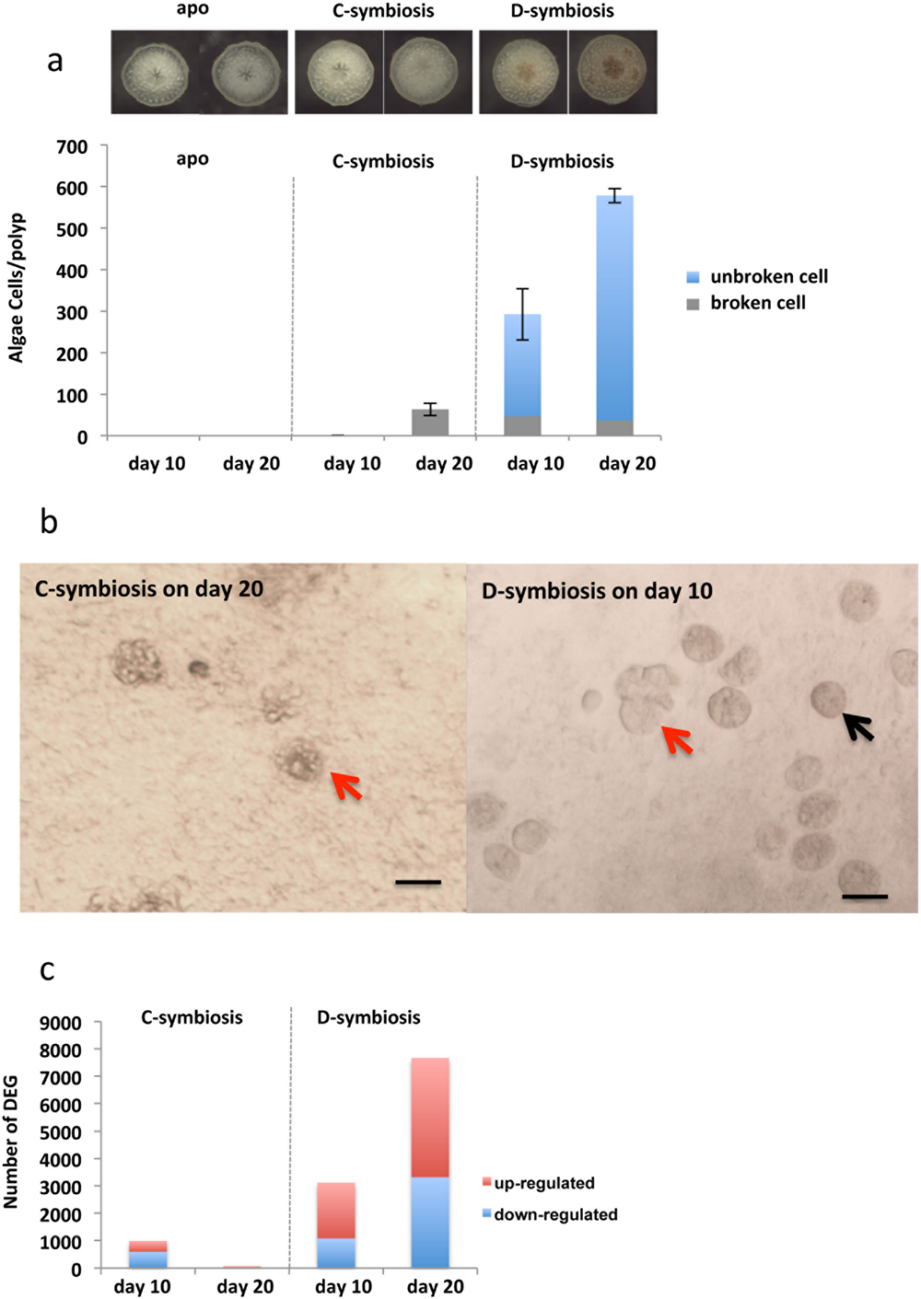
The early symbiotic stage of juvenile corals, and the number of differentially expressed genes. (**a**) Clade D *Symbiodinium* increased in *Acropora tenuis* juveniles (D-Symbiosis), whereas clade C colonization (C-Symbiosis) was very slow within 20 days. (**b**) Abnormally expanded *Symbiodinium* cells were observed in coral homogenates. Red arrows indicate expanded *Symbiodinium* cells and black arrow indicates normal *Symbiodinium* cell. Scale bars = 0.01 mm. (**c**) Bar graph showing the number of differentially expressed coral genes detected at days 10 and 20 after inoculation with *Symbiodinium* based on comparisons with aposymbiotic corals (apo).

To provide corals in the early symbiosis stage for RNA-seq analyses, *A*. *tenuis* juvenile corals (corals a few weeks after settlement) were maintained in artificial seawater, with subsequent introduction of *S. goreaui* (clade C) and *S. trenchii* (clade D) [hereafter, corals inoculated with *S. goreaui* (clade C) are described as “C-corals”; those inoculated with *S. trenchi* (clade D) are described as “D-corals”]. The establishment process of the coral–algal endosymbiosis was observed using a stereoscopic microscope for the first 20 days after *Symbiodinium* inoculation (Fig. 1a). Additionally a coral homogenate was observed under a microscope and the number of endosymbiotic algae counted (Fig. 1b). C-corals had almost no algae after 10 days (n = 10), with the average number of clade C cells per polyp at 20 days being 63.3 ± 15.4 (mean ± SE, n = 7). However, all of the clade C endosymbionts were abnormally shaped (swollen or fragmented) (Fig. 1b). Clade D cells in the polyps numbered 292.4 ± 61.6 (n = 5) at 10 days, increasing to 578.3 ± 17.4 (n = 6) by 20 days. Abnormal shapes were observed in 16.4% of the clade D symbionts at 10 days and 11.4% of symbionts at 20 days (Fig. 1b). In past studies, abnormally broken algal cells have been observed in histological sections of primary polyps^47^ and adult corals exposed to higher temperature^48^, indicating that symbiotic algae could be digested in host cells, especially at the onset of endosymbiosis and under bleaching conditions.

In contrast to clade C, clade D densities showed a relatively steady increase, which may be attributable to their ability to tolerate or avoid the host’s immune system^49^. Transcriptome analysis of these corals is one of the most effective ways of determining the nature of changes that occur with the success and failure of endosymbiosis.

### Increasing numbers of differentially-expressed coral genes with increasing endosymbiont density

Corals were fixed 10 and 20 days following inoculation with the two *Symbiodinium* clades (Fig. 1a), for the RNA-seq analyses, performed using an Illumina Hiseq 2000 (Illumina Inc., San Diego, CA, USA). *De novo* transcriptome assembly from the Illumina sequencing data and Basic Local Alignment Search Tool nucleotide database (BLASTn) searches resulted in 108,247 contigs obtained from *A*. *tenuis*, and 22,773 and 22,426 from clade C and clade D *Symbiodinium*, respectively.

The GC content distribution of the assembled coral and *Symbiodinium* transcriptomes showed two clear peaks at approximately 43% and 55%, apparently having originated from the coral and symbiont, respectively, since the reference transcriptome data from the coral and algae showed similar GC contents (Supplementary Fig. S2). Gene expression was detected in the coral by mapping the data on a fasta file derived from corals, and the differentially expressed genes (DEGs) between symbiotic and aposymbiotic corals identified using a R package, edgeR (Fig. 1c). Reproducibility among biological replicates was relatively high (correlation coefficient >0.89), although it is necessary to pay attention to the fact that two biological replicates in each condition have insufficient statistical power to detect DEGs.

We identified 991 DEGs in C-corals at 10 days, 592 DEGs in C-corals at 20 days, 3,116 DEGs in D-corals at 10 days, and 7,667 DEGs in D-corals at 20 days (Supplementary Fig. S3). Venn diagrams (Supplementary Fig. S4) revealed overlaps among the four different biological conditions, but identified only three downregulated DEGs [three homologs of green fluorescent protein - (GFP)-like chromoproteins] that were common to all four (Supplementary Table S1), suggesting that coral GFP is more sensitive to contact with symbiotic algae at the onset of endosymbiosis. In order to investigate coral gene expression change correlated with algal density, correlation coefficients (R) between algal density (apo, 10 days, 20 days) and gene expression was calculated for D-corals. The results indicated 919 genes positively correlated (R > 0.90) and 405 genes negatively correlated (R < −0.90) with algal density. Tables S2 and S3 show the 100 genes most highly correlated (R > 0.90, or R < −0.90) with algal density (cells/polyp) and gene expression. Genes for peroxidasin, glutamine synthetase and solute carrier family 26 member 6 (SLC26A6) had a positive slope, indicating they were strongly up-regulated by the increase in algal cell density (Supplementary Table S2). In contrast, genes for Alpha-glucosidase, low-affinity immunoglobulin epsilon Fc receptor and programmed cell death protein, for example, had a steeply negative slope, indicating their strong down-regulation with increasing symbiont density (Supplementary Table S3).

The RNA-seq method was validated using quantitative polymerase chain reaction (qPCR) and the gene expression of 13 randomly selected DEGs compared (Supplementary Fig. S4). The expression patterns observed in the qPCR results were generally consistent with the patterns obtained by RNA-seq. In addition, algal gene expression was also detected from the endosymbionts. Due to a lack of control conditions for *Symbiodinium*, the detection of DEGs using the *Symbiodinium* transcriptome could not be statistically analyzed. However, all *Symbiodinium* genes expressed in each sample were isolated and utilized for the discussion of *Symbiodinium*-coral interactions. Among the 22,773 clade C contigs and 22,426 clade D contigs, 48.3% and 71.1%, respectively, showed significant similarities (Blastx e-value < 1e–4) to the Swiss-Prot database.

**Figure 2:**
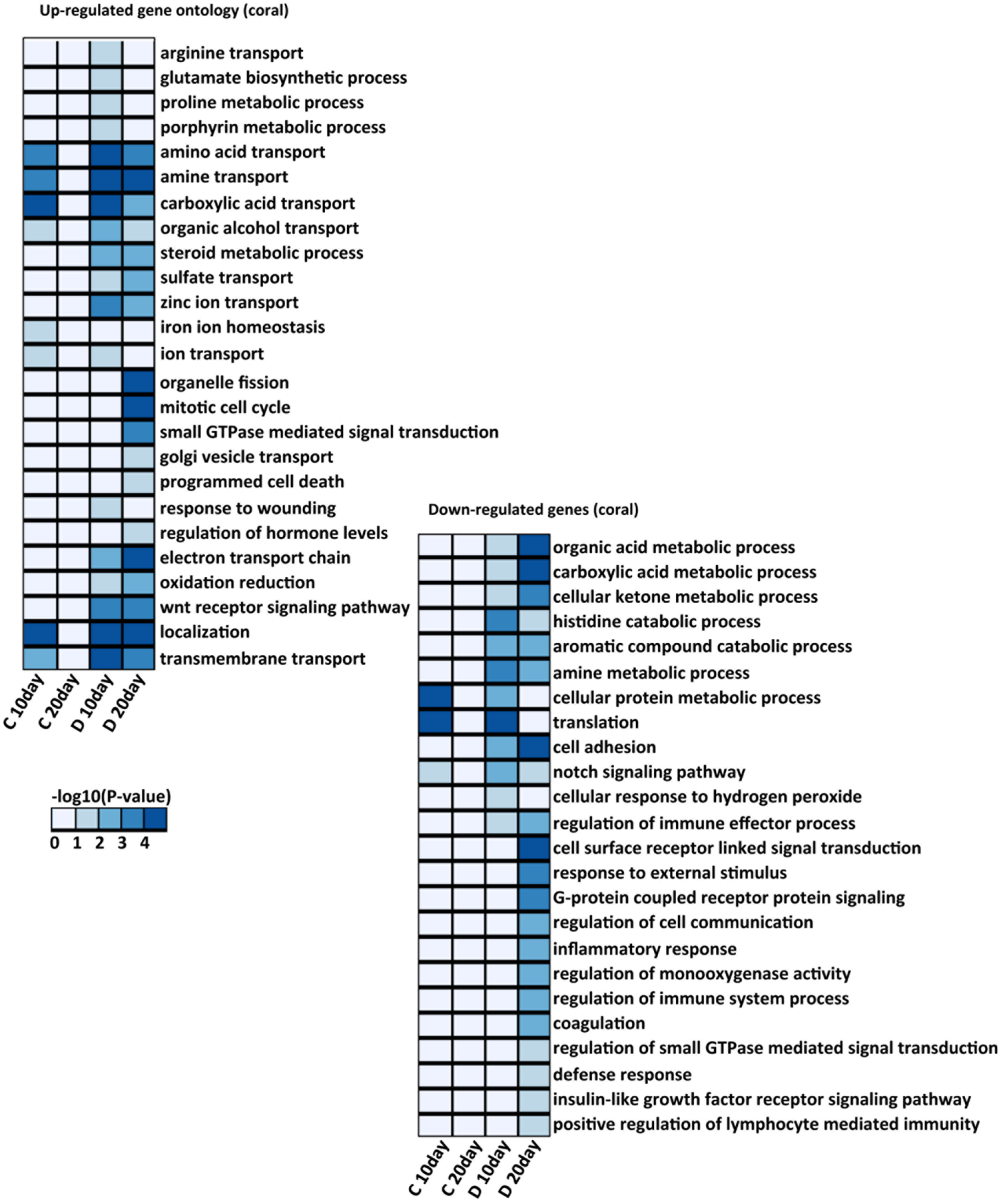
Heat map showing the p-value significance of enriched GO categories in up-regulated and down-regulated genes in 10 days and 20 days after inoculation of clade C and D *Symbiodinium*. The color intensities indicate the (−log 10(p-value)) enrichment score of each GO term.

Gene Ontology (GO) enrichment and Kyoto Encyclopedia of Genes and Genomes (KEGG) pathway analyses were performed using DAVID (https://david.ncifcrf.gov/)^50^ and the KEGG mapper (http://www.genome.jp/kegg/mapper.html)^51^ to identify enriched biological processes from the coral DEGs and algal transcriptome. Fig. 2 shows the GO annotation results for up and downregulated genes. The results showed that the upregulated genes in D-corals included a large number involved in metabolism, indicating that the presence of symbiotic algae has a great influence on coral metabolism. The C-corals, on the other hand, had a considerably smaller number of DEGs, with only a few enriched GO and KEGG pathways being detected. The results of the D-coral analysis provide the basis of the following discussion on the establishment of endosymbiosis, and are compared with the clade C results representing a case in which algae do not increase in density.

### Transition of the coral immune system to accept algal endosymbionts

A number of immune system-related genes were detected as symbiosis-associated genes. GO terms associated with regulation of the immune effector process, defense response, immune system processes, and positive regulation of lymphocyte-mediated immunity were downregulated in D-corals (Fig. 2 and Supplementary Fig. S4). These GO terms include tumor necrosis factor receptor-associated factor 6, matrix metalloproteinase 9, low-affinity immunoglobulin epsilon Fc receptor, lipopolysaccharide binding protein, and toll-like receptor 2. The decrease in expression of gene encoding low-affinity immunoglobulin epsilon Fc receptor showed a strongly negative correlation with increasing clade D algal cell density, suggesting that a decrease in the gene expression may be caused by the link to algal cell density (Supplementary Table S3). In this context, genes related to the TGFβ signaling pathway were also upregulated in D-corals (TGFβ acting as a key regulator of immune tolerance and inflammatory responses) (Supplementary Fig. S5c). In *Aiptasia*, adding anti-TGFβ to block the putative TGFβ pathway results in immune stimulation and a failure of the symbiont to colonize the host^28^. Such changes in the host immune system could be important for maintaining symbionts in the host cells, as well as during viral and bacterial infections^52^. In the case of C-corals, such changes relating to immune system reduction were not detected. The different expression patterns of the host immune system depended on the symbiont type, revealing that a decline in the immune system is necessary for the acceptance of many symbionts into host tissue.

### Activation of endocytosis and deactivation of lysosomal acid hydrolase

**Figure 3:**
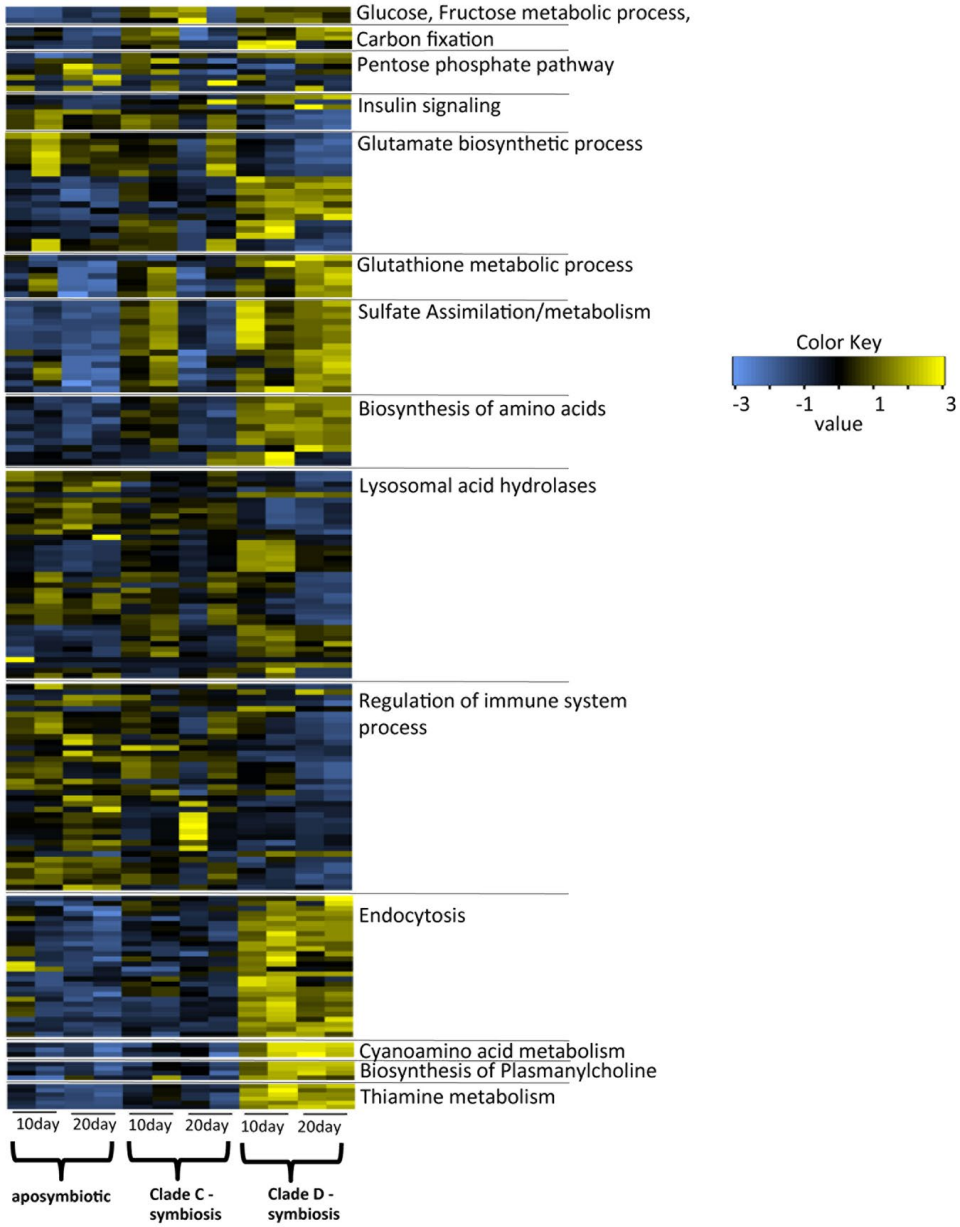
Heatmap of differentially expressed genes (DEGs) in aposymbiotic corals (aposymbiotic), corals associated with clade C (clade C-Symbiosis) and those associated with clade D (clade D-Symbiosis). There are selected notable treatment-specific genes (FDR P < 0.05) form each functional categories. The color shows expression levels normalized to z-score in all biological replicates; blue and yellow colors denote low and high intensities, respectively. The original heatmaps of each functional category are shown in Supplementary.

The results of the KEGG pathway analysis revealed dynamic changes in the endocytosis pathway in D-corals (Supplementary Fig. S5abc). Clathrin-dependent endocytosis, as well as recycling of endosome- and late endosome-associated proteins, were upregulated with an increase in the number of algae. Since the clathrin-mediated pathway of endocytosis has an upper size limit for internalization of approx. 200 nm, it could not be directly associated in the incorporation of endosymbiotic algae, but be associated in a recycling of symbiosome membrane^53,54^. Our results showed that the expression levels of Rab GTPase family proteins (Rab proteins) 7, 8 were changed significantly in D-corals (Fig. 3, Supplementary Fig. S6a). Studies of *Aiptasia* have demonstrated that several types of Rab proteins (Rab 3, 5, 7, and 11) are localized in the symbiosome, each playing an important role in the maintenance, degradation and recycling of endosymbionts^25–27^. These studies show that Rab 7 is absent in the majority of phagosomes containing live and healthy zooxanthellae in *Aiptasia*, whereas phagosomes containing heat-killed or -damaged zooxanthellae stain positively for Rab 7^25^. In corals, Rab 7 was reported to be expressed at significantly higher levels under high-light/high-temperature conditions associated with oxidative stress and coral bleaching^55^. Accordingly, Rab 7 may be involved in the elimination of damaged *Symbiodinium*. Another upregulated Rab protein, Rab 8, is a membrane trafficking protein with functions in the membrane recycling process^56^. The high expression of these Rab proteins in D-corals may be associated with eliminating broken algal cells found during the early stages of endosymbiosis (Fig. 1b).

A large number of Rab proteins were also identified in the algal transcriptome, including Rab 1, 2, 3, 4, 5, 6, 7, 11, 23 and 28 (Supplementary Fig. S6b). Among them, Rab 5b is a candidate symbiosis-related algal gene, having been identified from the *Plasmodium* parasite, where it was localized in the parasitophorous vacuole membrane surrounding intracellular *Plasmodium*^57^. In the case of the symbiosome membrane of a cnidarian host, the symbiosome is partially derived from *Symbiodinium* itself^58^. The identification of Rab 5b from *Symbiodinium* suggests the possibility that S*ymbiodinium-*derived Rab proteins may also become localized in the symbiosome membrane, being involved in the establishment of endosymbiosis.

Although some endocytosis-related genes were expressed at greater levels in D-corals, lysosomal acid hydrases, such as proteases, lipases, sulfatases, glucosidases, sphingomyelinases and ceramidases, tended to decrease (Fig. 3, Supplementary Fig. S7). In particular, downregulation of several digestive enzymes targeting glycan is possibly related to the establishment of endosymbiosis. Because glycan is a cell wall component of *Symbiodinium*^59^, such declines in digestive enzymes would seem essential to avoid the digestion of endosymbiotic *Symbodinium* cells. Our observations indicated that symbiotic algal cells were broken 10 days after inoculation, the proportion of broken cells thereafter having decreased at 20 days. Such a decrease was probably associated with a decrease in the expression of digestive enzymes in the corals.

### Nonsense transcripts expressed in the symbiont

As mentioned above, a decline in the coral immune system and digestive enzyme activities occurs at the beginning of endosymbiosis, although the trigger for such transcriptome changes remains unclear. However, examining characteristic categories from the *Symbiodinium* transcriptome provides an insight, for example, of genes involved in microRNA expression in *Symbiodinium*. A gene cluster associated with RNA interference, including a regulator of the nonsense transcript 1 homolog and a probable ATP-dependent RNA helicase spindle-E, was enriched in symbiotic clade D *Symbiodinium* on day 20 (Supplementary Fig. S8). This suggests that nonsense-mediated RNA decay occurred in the symbiont as density of the latter increased and that symbiont nonsense RNA modulates the cnidarian host transcriptome, including immune system-related and digestive enzyme-related genes. Some studies have suggested that cnidarian protein-coding genes are predicted targets of symbiont miRNA*-*mediated post*-*transcriptional regulation^42,60^. In plant root endosymbioses (e.g., the legume *Rhizobium* with arbuscular mycorrhizal fungi) and human bacterial infections, symbiont-derived miRNA sequences significantly regulate the expression of targeted host genes^61^. Thus, although further verification of the function of miRNA in endosymbiosis is needed, RNAi via miRNA may well be important in the intracellular symbiotic system.

### Changes in metabolic processes

The transcriptome data revealed that coral metabolism increased dramatically as a result of the increase in the number of *Symbiodinium* in corals (Figs 2,3). The pathways related to carbon metabolism and biosynthesis of amino acids were enriched as the genes encoding their components were upregulated in corals colonized with a high density of clade D *Symbiodinium* (Fig. 3, Supplementary Figs S9, S10). Upregulation of coral carbon metabolic processes is closely related to the photosynthetic pathway of *Symbiodinium*. *Symbiodinium* possesses the crassulacean acid metabolism (CAM) photosynthetic pathway^62^, which largely influences coral metabolism because genes related to the metabolism of pyruvate, malate, and oxaloacetate, the CAM photosynthetic metabolites, were upregulated in symbiotic corals (Supplementary Fig. S11). The expression of genes related to fructose metabolism, such as the fructose-1,6-bisphosphatase gene, increased remarkably (Supplementary Fig. S11), indicating that corals obtain a large amount of fructose from algae. One of the glucose metabolic pathways, the pentose phosphate pathway, was also upregulated in D-corals (Supplementary Fig. S12). The pentose phosphate pathway and its intermediate products are involved in the production of many bioactive substances, including nucleotides and MAAs, which are UV-absorbing molecules^63^. These results revealed that coral could receive source of carbon hydrate and nucleic acids from symbiont.

The existence of glucose metabolism regulating systems was also suggested by the transcriptome data obtained here. A series of insulin signaling pathways linked to lipogenesis, glycogenesis, anti-lipolytic functions, insulin-like growth factor and insulin-like growth factor receptor homologs were up- or down- regulated in D-corals (Figs 2,3 and Supplementary Fig. S13). Although such signaling pathways have not yet been elucidated in corals, insulin plays an important role in regulating developmental processes in *Hydra*^64,65^. Additionally, *Symbiodinium* may also be indirectly affected by insulin-like growth factor, since genes encoding insulin-induced or insulin-degrading enzymes were detected among the *Symbiodinium* transcripts. Although further confirmation of the presence of insulin-like substances is necessary, our data suggested that such substances could regulate coral glucose metabolism, since a series of genes related to insulin were detected in both coral and zooxanthella. Considering that *Symbiodinium* provides glucose to corals^11^, the coral insulin signaling pathway may have a role in restricting the use of sugars obtained from the algae.

*Symbiodinium* also possesses a pathway for ammonia assimilation into glutamate, such metabolic processes facilitating the effective use of amino acids in corals (Figs 2,3, Supplementary Fig. S14). Glutamine synthesis and related metabolic processes, such as proline metabolism, were upregulated in D-corals at 10 days, and it appears that corals promote the metabolism of glutamine (Supplementary Fig. S15). The results were similar to past studies involving RNA-seq analysis of algal-symbiotic and non-symbiotic anemones, which reported that transcripts for glutamine synthetase and an NADPH-dependent glutamate synthase are upregulated in the symbiotic state^50^. Proline synthetic pathways of corals were also enhanced in D-corals and their symbiont (Supplementary Fig. S15). Proline mignt be transported to endosymbiotic algae because osmoregulated proline transporters are expressed in endosymbiotic algae. As the transporter name implies, proline is a common osmolyte in plants and bacteria^66^. Some osmoregulated proline transporters were expressed in clade D symbionts, but not in those of clade C, suggesting that proline is necessary for algae to survive in host cells under higher-osmolality conditions. The results further showed that glutathione peroxidase, which catalyzes the reduction of hydroxyperoxides by glutathione, is expressed at higher levels in D-corals than in C-corals (Supplementary Fig. S16). Glutathione is synthesized from glutamate, the metabolism of which is activated by algal symbiosis. Hence it is possible that glutathione peroxidase utilizing glutamate may be activated in symbiotic corals. Glutathione peroxidase acts as a stress marker gene in corals, its expression increasing under stressful conditions^67^. Because symbiotic *Symbiodinium* itself produces reactive oxygen species, especially under high temperature and light stress, corals are generally at risk of oxidative stress by acquiring algae. The ability to modulate antioxidation by enzymes, such as glutathione peroxidase, is indispensable for accepting endosymbiotic algae, the synthesis of such enzymes being activated by the algae.

In addition, sulfur assimilation was also upregulated by the clade D symbiont (Fig. 3, Supplementary Fig. S17a). As past studies have demonstrated, sulfate utilization by corals may be influenced by algal endosymbiosis, the up-regulation of the sulfate assimilation process possibly being a consequence of algal-coral endosymbiosis^68^. The sulfate assimilation related enzyme bifunctional 3′-phosphoadenosine 5′phosphosulfate synthase underwent increased expression in D-corals, but the homologous gene could not be detected in *Symbiodinium*, which instead had a gene encoding the adenosine 3′-phospho 5′-phosphosulfate transporter (Supplementary Fig. S17b). It is therefore conceivable that corals with algal cells actively uptake sulfate ions and synthesize Adenosine 3′-phospho 5′-phosphosulfate, which is transported to *Symbiodinium*.

Other metabolic processes enhanced by algal symbiosis include biosynthesis of the neurotoxins^69^, *γ-glutamyl-β*-cyanoalanine and *γ-glutamyl-β-aminopropionitrile*, biosynthesis of plasmanylcholine and metabolism of thiamine (vitamin B1). Plasmanylcholine is involved in acclimation to a temperature-fluctuating environment in Screlactinian corals^70^ (Fig. 3, Supplementary Figs S18–S20). Although most corals cannot alone synthesize these substances, they may be synthesized using metabolites of the symbiont, and accelerated synthesis of these substances could affect in coral health and defense mechanism.

**Figure 4:**
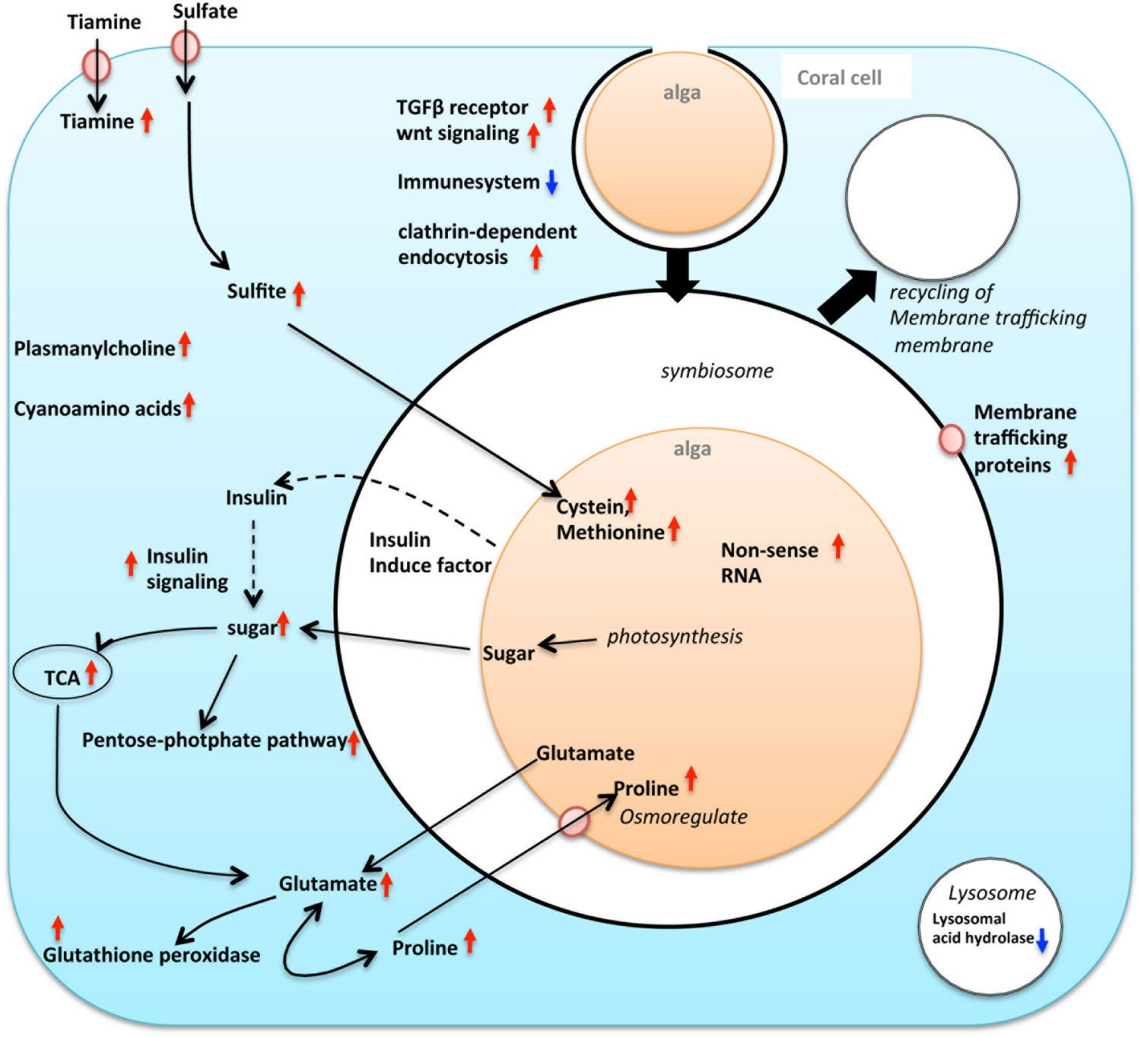
Schematic summary of events presumably occurring in the early symbiosis of reef-building corals and alga, based on the transcriptome data of D-corals. Red arrows indicate up-regulated genes, and blue arrow indicate down-regulated genes with with increasing algal symbiont. The metabolic pathway and signaling pathway influenced by the presence of algal symbionts are represented by thin black arrows and dashed arrows, respectively.

Taking our findings together, we detected the processes required to establish endosymbiosis by focusing on transcriptomic changes during the early stage of symbiosis (Fig. 4). The following processes were considered to occur during the establishment of symbiosis: 1) Upregulation of the endocytosis pathway and a decrease in the expression of digestive enzymes for uptake and maintenance of algal symbionts. 2) The coral immune response system was partially inactivated. 3) miRNA of the endosymbiotic algae was overexpressed during the early stage of endosymbiosis. Additionally, the following coral metabolic processes changed dramatically: metabolism of sugar, pyruvate, malate, oxaloacetate, glutamate, thiamine and proline, and biosyntheses of *γ-glutamyl-β*-cyanoalanine*, γ-glutamyl-β-aminopropionitrile*, plasmanylcholine. The transcriptome data also suggested that corals regulate sugar metabolic processes via an insulin-like signaling pathway.

In this study, we clarified the endosymbiosis process by comparing two algal symbiotic systems with different rates of increase in the number of symbionts. The study investigated endosymbiosis establishment based on corals colonized by *Symbiodinium trenchi* (clade D). Accordingly, it is necessary to verify whether or not the symbiosis-related changes described here would occur generally in response to other *Symbiodinium* clades. Notwithstanding, the changes in expression of host genes with an increased density of symbiotic algae is expected to further clarify our understanding of endosymbiosis-related pathways and metabolism.

## Materials and Methods

### Animals and algae

*Acropora tenuis* were collected around Akajima Island, Okinawa by Akajima Marine Science Laboratory (Okinawa, Japan). Collection of *A. tenuis* larvae was performed as previously described in Iwao *et al*.^71^. Several days after spawning, metamorphosis was induced by exposure of larvae to 2 μM Hym 248 in containers (55-mm diameter)^34^. The *Symbiodinium* strains CCMP2466 (clade C1) and CCMP2556 (clade D) were obtained from the Bigelow Laboratory for Ocean Sciences (West Boothbay Harbor, ME, USA; https://ccmp.bigelow.org/) and cultured in f/2 medium (Wako Chemicals, Osaka, Japan) with antibiotics (kanamycin 20 μg/mL and ampicillin 50 μg/mL) at 24 °C under a 12-h light (20 µE/m^2^/s), 12-h dark cycle. Juvenile polyps were cultured in Petri dishes containing artificial seawater (KUMARINE SeaSalt α, Marine route one, Kanagawa, Japan) at 24 °C under a 12-h light (70 µE/m^2^/s), 12-h dark cycle. Cells of each strain of *Symbiodinium* algae (approximately 1,000 cells per polyp) were introduced to *A. tenuis* primary polyps 10 days after settlement. Each *Symbiodinium* culture was subsequently introduced into Petri dishes containing polyps every day. Approximately 40 larvae settled in each dish. Some of the juvenile polyps were maintained in an aposymbiotic state and used for experiments. Five dishes were used for each treatment (inoculation with clade C1, inoculation with clade D, aposymbiotic), and seawater was changed daily.

### Microscopic observations

Juvenile polyps were observed with a stereomicroscope during the incubation period. Color micrographs of several polyps were taken with a digital scanning microscope (model VHX-1000; Keyence, Tokyo, Japan) using a digital camera (Digital Slight DA-L1; Nikon) to evaluate the density of symbionts. Micrographs of polyps were taken 10 and 20 days after inoculation with *Symbiodinium* algal cultures. Approximately five polyps were photographed in each treatment.

### *Symbiodinium* cell counts

Polyps were fixed in 3% formaldehyde 10 and 20 days after inoculation with *Symbiodinium*. Subsequently, samples were decalcified using decalcification solution containing 0.5 M ethylenediaminetetraacetic acid (EDTA) for 2 days^36^. Each polyp was placed in a 1.5-mL tube containing 0.01% TritonX, and homogenized. Algal cells in the homogenate were counted using a hemocytometer (Thomas Scientific, Swedesboro, NJ). Three or four polyps were used for counting of *Symbiodinium* algal cells in 10 days and 20 days after inoculation with *Symbiodinium*. Polyps associated with clade C1 algae contained a small number of *Symbiodinium* cells. These polyps were crushed using a cover glass onto a glass slide to count their endosymbiotic *Symbiodinium* cells, because a very small number of algal cells could be lost in the homogenate and hemocytometer.

### RNA extraction

Polyps collected 10 and 20 days after inoculation with clade C or clade D *Symbiodinium* cultures were fixed in RNAlater (Ambion, Austin, TX, USA). Polyps in four replicates of each treatment were fixed and used for RNA extraction, and one or two replicates were used for each set of biological observations. For RNA-seq analysis, two biological replicate were prepared for each day (one replicate derived from one petri dish). Total RNA was extracted from juvenile polyps using a PureLink RNA Mini kit (Life Technologies corporation, Carlsbad, CA). The total RNA was treated with DNase I (TAKARA, Ohtsu, Japan) to digest genomic DNA, and then mRNA was purified from the samples using the NEBNext Poly(A) mRNA Magnetic Isolation Module (NEB, Ipswich, MA).

### RNA-seq

cDNA libraries were generated from mRNA samples using the NEBNext mRNA Library Prep Master Mix Set for Illumina (NEB). Pared-end sequencing of 100 bp was performed by Macrogen Japan using a HiSeq 2000 sequencer (Illumina, San Diego, CA), which resulted in over 35 million reads per sample. Short reads were first pre-processed, trimming bases with a Phred quality score below Qv = 20 from the 5′ end and 3′ end of each read, and retaining reads ≥25 bp, while reads with 30% of bases having Qv ≤ 15 were filtered out. This processing was performed using the DDBJ pipeline (https://p.ddbj.nig.ac.jp/pipeline/Login.do). All sequence data were deposited in the DDBJ/EMBL/GenBank databases under accession number DRA006413.

### Identification of differentially expressed genes between coral samples

Trinity (version, 2.1.1) software was used to assemble *de novo* transcripts from all reads in 12 *A. tenuis* samples (aposymbiotic corals, clade C symbiotic corals, and clade D symbiotic corals). Then, TransDecoder (http://transdecoder.sourceforge.net/) was used to identify candidate regions from the assembled transcripts or contigs. To detect contig sequences originating from the host coral, we built custom coral and *Symbiodinium* databases. The coral database included an *A. digitifera* genome^40^, non-symbiotic *A. hyacinsus* and *A. tenuis* transcriptomes (http://www.bio.utexas.edu/research/matz_lab/matzlab/Data.html), and *de novo A. tenuis* transcriptomes obtained from aposymbiotic polyps in this study. The *Symbiodinium* database includes a *Symbiodinium* genome^41^, and *Symbiodinium* clade C and clade D transcriptome data from the PALUMBI Lab^72^. The nucleotide sequences of the assembled contigs were aligned to each custom database using BLASTn, and contigs that aligned only to the coral database were annotated as *A. tenuis* contigs. Different e-value cutoffs (1e^−1^, 1e^−2^, 1e^−3^, 1e^−4^, 1e^−5^, and 1e^−10^) were examined and the cutoff value (1e^−4^) that maximized the number of *A. tenuis* contigs was adopted with reference to Shinzato *et al*.^73^. Reads were aligned to coral contigs using bowtie2 (version 2.2.4) software and mapped reads were counted using eXpress (version, 1.5.1) software. The R package edgeR was used to compare gene expression between inoculated polyps and non-treated polyps after 10 or 20 days, and to identify detected contigs as differentially expressed if the adjusted false-discovery rate was P ≤ 0.05.

### Analysis of the *Symbiodinium* tnranscriptome

To detect contig sequences originating from clade C or clade D *Symbiodinium*, a *de novo* transcript was assembled using Trinity (version, 2.1.1) from 522 million reads from 8 *A. tenuis* samples associated with clade C *Symbiodinium* and 493 million reads from 8 *A. tenuis* samples associated with clade D *Symbiodinium*. Then, TransDecoder was used to identify candidate regions, and contigs that aligned only to the *Symbiodinium* database were annotated as *Symbiodinium* clade C or clade D transcripts by the same method used for identification of coral genes. For analysis of *Symbiodinium* transcriptomes, reads from samples collected 10 and 20 days after inoculation were aligned to *Symbiodinium* contigs and counted using bowtie2 (version, 2.2.4) and eXpress (version, 1.5.1). Contigs that were aligned ≥1 time were annotated against the SwissProt protein database and NCBI non-redundant protein database (nr) using the blastx program (BLAST 2.2.30) to determine their functions.

Blastx search, GO enrichment analysis, and KEGG pathway analysis of DEGs, and quantitative PCR can be found in SI Materials and Methods.

## Electronic supplementary material

SI Materials and Methods, and Supplementary Figures S1-S20, Supplementary Table S1

Supplementary Table. S2

Supplementary Table. S3

Supplementary_csv
