# Supplementary material for: Transcriptomic changes with increasing algal symbiont reveal the detailed process underlying establishment of coral-algal symbiosis
Source: Sci Rep. 2018 Nov 14;8:16802. doi: 10.1038/s41598-018-34575-5 (PMC6235891; doi:10.1038/s41598-018-34575-5)
Supplement: Supplementary file 1 — SI Materials and Methods, and Supplementary Figures S1-S20, Supplementary Table S1 [file 41598_2018_34575_MOESM1_ESM.pdf]

Supplementary Information

Transcriptomic changes with increasing algal symbiont reveal the detailed process underlying establishment of coral-algal symbiosis

Ikuko Yuyama, Masakazu Ishikawa, Masafumi Nozawa, Masa-aki Yoshida, Kazuho Ikeo

Tale of Content

I. SI Materials and Methods

I.1 Blastx search, GO enrichment analysis, and KEGG pathway analysis of DEGs…………..3

I.2 Quantitative PCR….………………………………………………………..……...3

II. Supplementary Figures

Fig. S1. The distribution of GC contents of *Acropora, Symbiodinium* gene dataset……….…4

Fig. S2. Venn diagram showing overlap of differentially expressed coral genes as identified with RNA-seq. ……………………………………………………………..…….5

Fig. S3. Comparison of expression profiles obtained from RNA-Seq and qPCR analysis of the same samples. ……………………………………………………………...…….6

Fig. S4. Heat map of the expression patterns of genes related to Regulation of immune system process. ………………………………………………..…………….………7

Fig. S5abc. Gene expression patterns of endocytosis and lysosomal related genes in corals ……………………..…………………..……………………………………….8

Fig. S6ab. Heat map of the expression patterns of genes related to endocytosis..…………….....11

Fig. S7. Heat map of the expression patterns of genes related to lysosomal acid hydrolase….13

Fig. S8. Heat map of the expression patterns of genes related to RNAi…………………..….14

Fig. S9. Heat map of the expression patterns genes related to Glucose, Fluctose metabolic process. ………………………………………………..……………..…….15

Fig. S10. Heat map of the expression patterns genes related to biosynthesis of amino acids... 16

Fig. S11.Heat map of the expression patterns genes related to carbon fixation……………….16

Fig. S12. Heat map of the expression patterns genes related to Pentose phosphate pathway…17

Fig. S13. Heat map of the expression patterns genes related to Insulin signaling………….….18

Fig. S14. Heat map of the expression patterns of genes involved in nitrate assimilation. ...…19

Fig. S15. Heat map of the expression patterns of genes related to glutamate biosynthetic process.……………………………………..……………………………20

Fig. S16. Heat map of the expression patterns of genes related to glutathione metabolic

process..21
Fig. S17ab. Heat map of the expression patterns of genes related sulfur assimilation..........22
Fig. S18. Heat map of the expression patterns of genes related to cyanoamino acid metabolism. 23
Fig. S19. Heat map of the expression patterns of genes related to biosynthesis of plasmanylcholine..24
Fig. S20. Heat map of the expression patterns of genes related to thiamine metabolism. 26

III. Supplementary Table

Table. S1. Common differentially expressed genes (DEGs) in D-coral and C-coral on day10, day20. 27

Electronic supplementary material

- Table. S2. List of top 100 positive slope genes in the group with highly correlation coefficient (R>0.90) on relationship between algal density (cells/polyp) and gene expression (FPKM) of D-corals (apo, 10 days, 20 days).
- Table. S3. List of top 100 negative slope genes in the group with highly correlation coefficient (R<-0.90) on relationship between algal density (cells/polyp) and gene expression (FPKM) of clade D symbiosis polyp (apo, 10 days, 20 days).
- supprementary_csv. The results of blastx search of each contig against UniProtKB, and FPKM values of each contigs for all 12 samples
  (apo_10d_1, apo_10d_2: aposymbiotic corals replicate 1 and 2 at day 10,
  apo_20d_1, apo_20d_1: aposymbiotic corals replicate 1 and 2 at day 20,
  c_10d_1, c_10d_2: C-corals replicate 1 and 2 at day 10,
  c_20d_1, c_20d_2: C-corals replicate 1 and 2 at day 20,
  d_10d_1, d_10d_2: D-corals replicate 1 and 2 at day 10,
  d_20d_1, d_20d_2: D-corals replicate 1 and 2 at day 20).

I. SI Materials and Methods

I.1 Blastx search, GO enrichment analysis, and KEGG pathway analysis of DEGs

The contigs containing candidate coding sequences were BLASTX aligned against the NCBI nr protein database to evaluate sequence similarity to genes in other species with an E-value cutoff of 1e–04. To identify enriched functional gene groups and pathways, GO enrichment analysis was performed using the database for annotation, visualization, and integrated discovery (DAVID). Significant processes were selected based on a corrected P-value using the method of Benjamini with a cutoff of 0.05. Pathway enrichment analysis was also conducted using KEGG.

1.2 Quantitative PCR

To verify the quantitative accuracy of RNA-seq data, we investigated the expression levels of selected DEGs using qPCR. Total RNA samples extracted from clade D-colonized corals and aposymbiotic corals after 20 days were used for qPCR (same RNA samples with RNA-seq). cDNA were synthesized from the 50 ng total RNA with SuperScript VILO MasterMix (Invitrogen, Carlsbad, CA). Primer were designed using primer3plus (http://www.bioinformatics.nl/cgi-bin/primer3plus/primer3plus.cgi/) for contigs which showed up-regulated in clade D infected polyp. The quantitateive qPCR was performed in triplicate using SYBR Premix Ex Taq II (Takara) and the mean values were calculated. The data were analyzed using an Chromo 4^{TM} System (Bio-Rad, Hercules, CS, USA) with reaction conditions as follows: 95℃ for 10 min, 40 cycles of 95℃ for 15 sec and 60℃ for 60 sec. A ten-fold serial dilution (1 to 0.0001 ng) was prepared and used to generate a standard curve. Each standard curve was generated by linear regression of the plotted points. Three putative housekeeping genes were tested for the stability of their expressions using Normfinder software (http://moma.dk/normfinder-software) (Andersen et el., 2004). Between aspoymbiotic corals and symbiotic corals, the most stable expression was noted for β-tubulin. The most stable gene, β-tubulin, was used to calculate the normalization factor for each of the cDNA samples.

Reference for SI Materials and Methods**:**

Andersen CL, Ledet-Jensen J, Ørntoft T (2004) Normalization of real-time quantitative RT-PCR data: a model based variance estimation approach to identify genes suited for normalization - applied to bladder- and colon-cancer datasets. Cancer Res. 64:5245-5250

II. Supplementary Figures

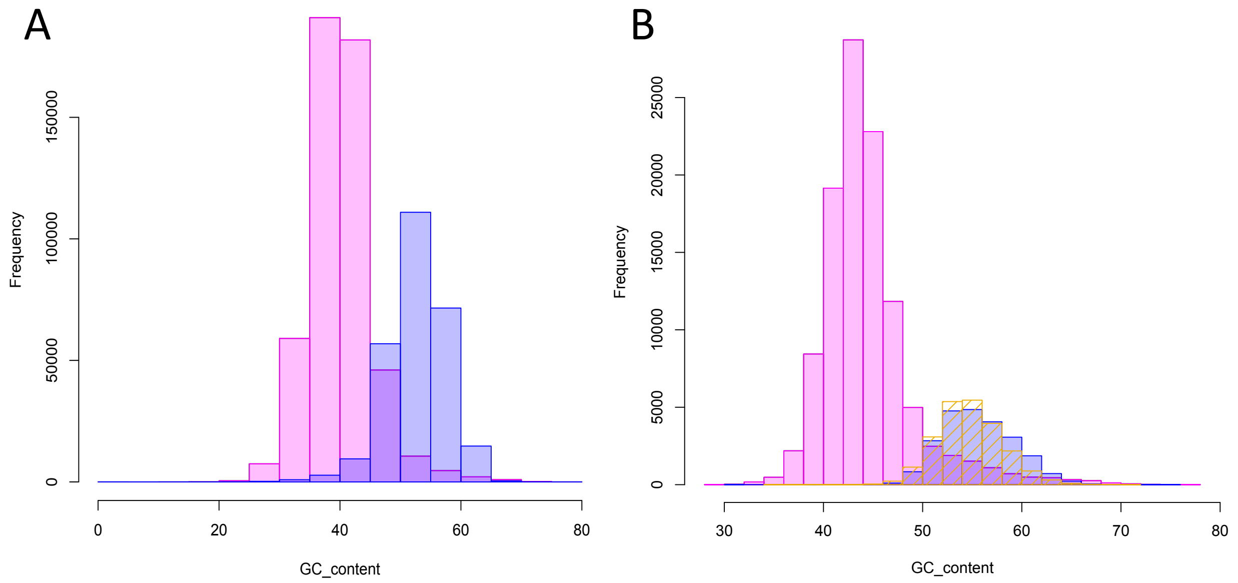

Fig. S1. The distribution of GC contents of *Acropora* , *Symbiodinium* gene dataset.
The distribution of GC contents of *Acropora* , *Symbiodinium* reference gene dataset (A), and GC contents of our assembled contigs from Illumina reads (B). Pink bars shows GC contents of *Acropora* data and blue r bars shows GC contents of *Symbiodinium* reference data. *Symbiodinium* dataset have a higher GC-contents in contrast to the Acropora dataset.

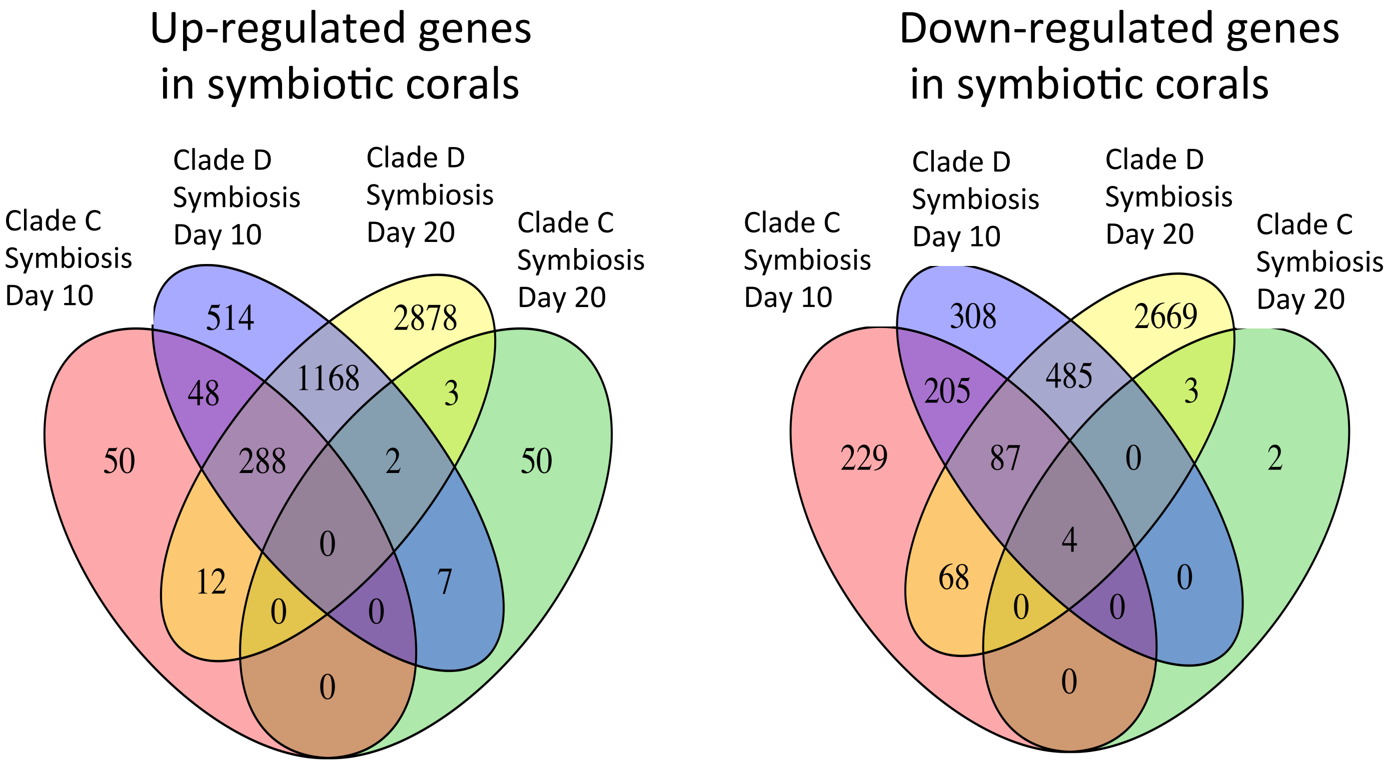

Fig. S2. Venn diagram showing overlap of differentially expressed coral genes as identified with RNA-seq.

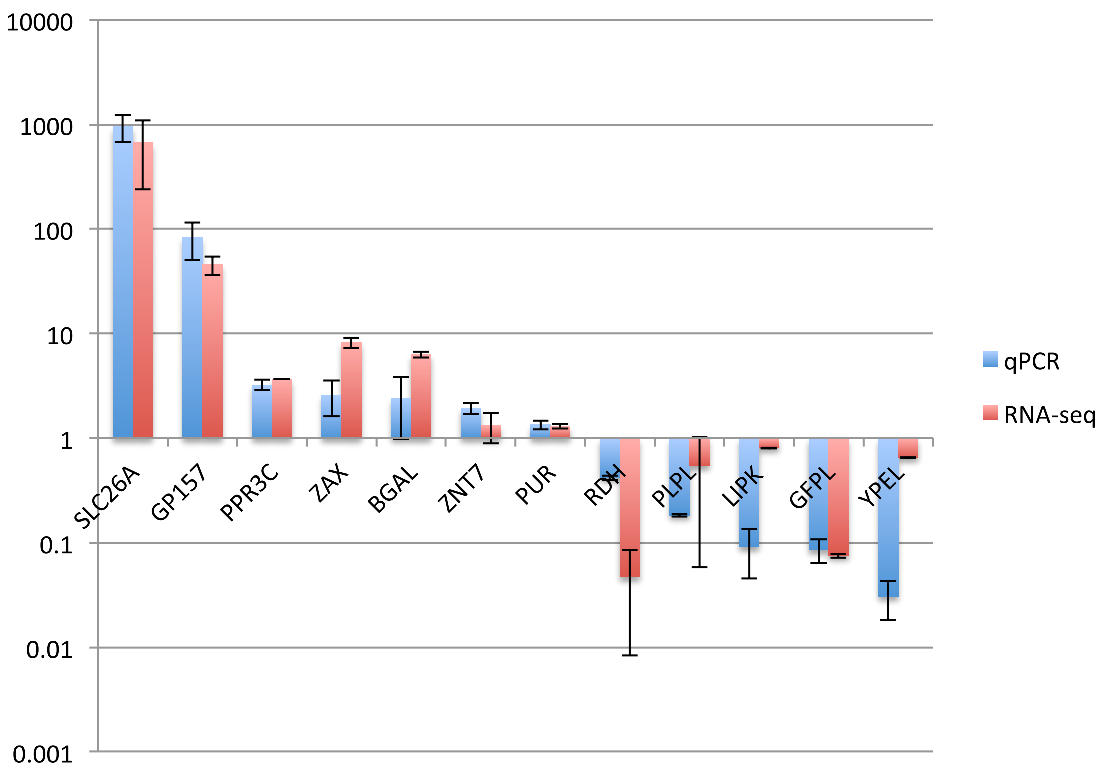

Fig. S3. Comparison of expression profiles obtained from RNA-Seq and qPCR analysis of the same samples.

Expression levels of 13 selected differentially expressed gene (DEG) identified by RNA-Seq was measured by qPCR . The average fold difference of each gene expression in corals associated with clade D as compared with aposymbiotic is presented for the average of 2 biological replicates in RNA-seq and three technical replicates in qPCR.

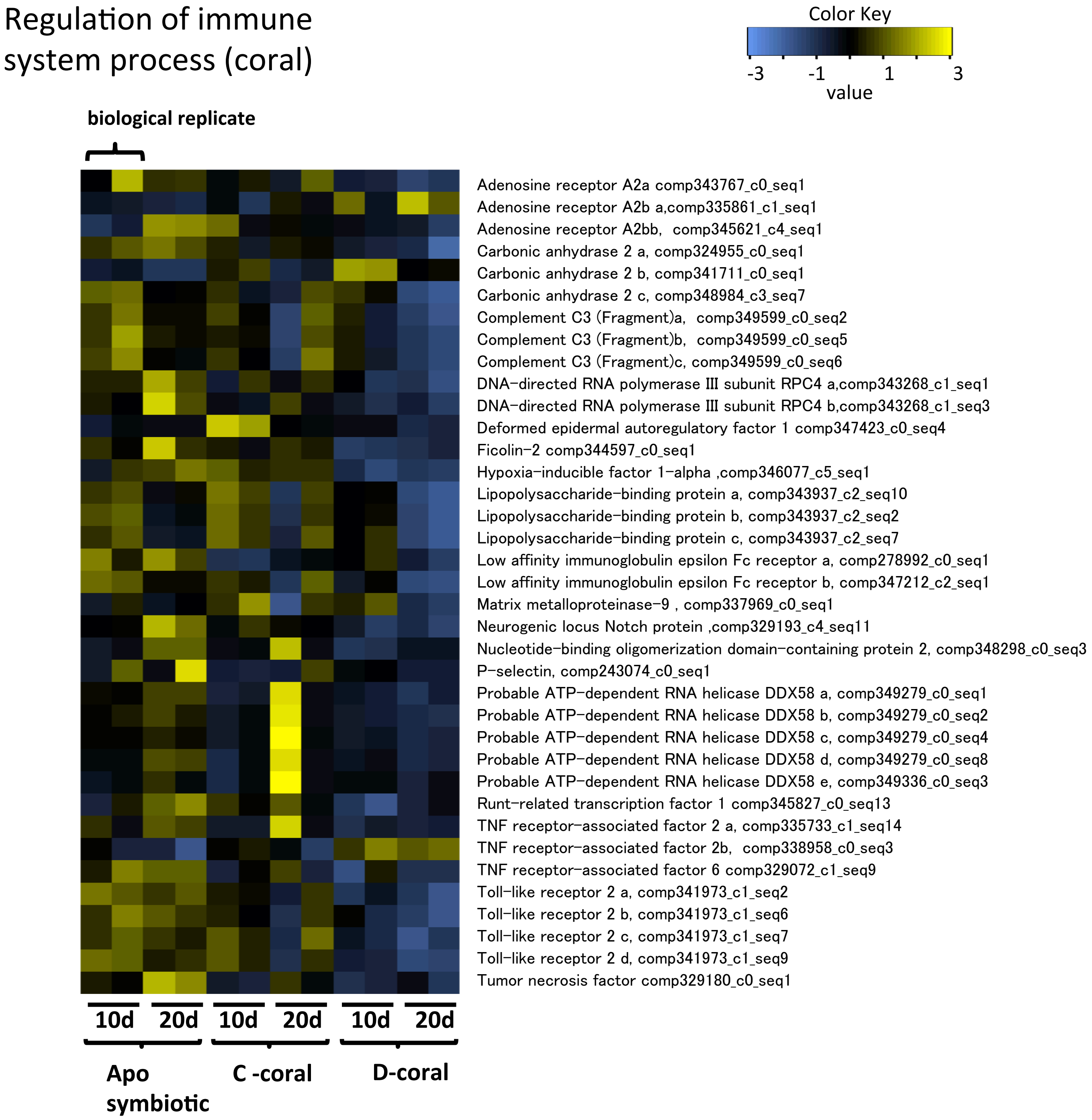

Fig. S4 . Heat map of the expression patterns of genes related to Regulation of immune system process.

Heat map shows the expression patterns of genes related to Regulation of immune system process in aposymbiotic corals, corals associated with clade C (C-coral) or clade D (D-corals) in 10 days and 20 days after inoculation of each *Symbiodinium*. There are selected notable treatment-specific genes (FDR P < 0.05).

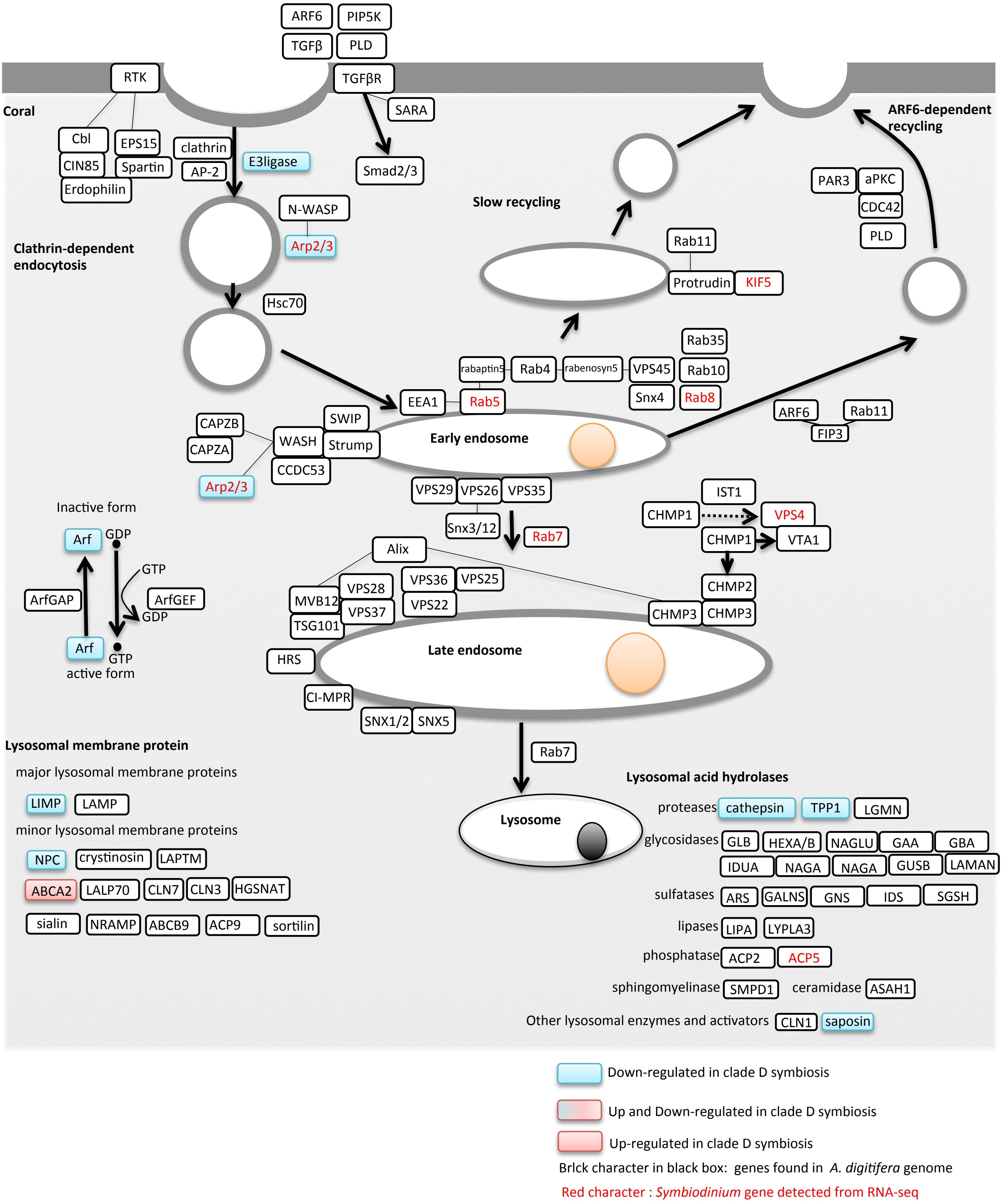

Fig. S5a. Gene expression patterns of endocytosis and lysosomal related genes in corals associated with clade C on 10days.

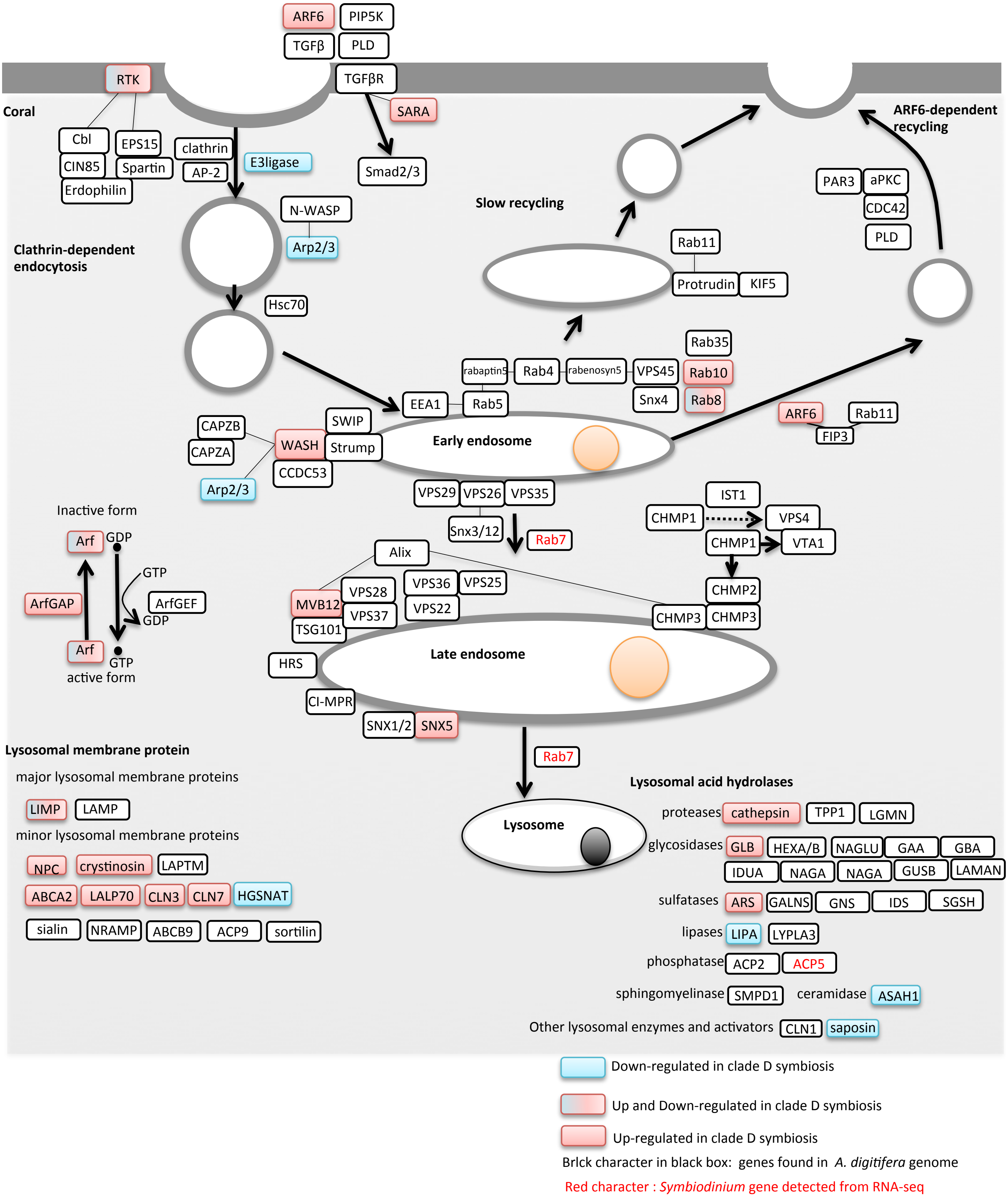

Fig. S5b. Gene expression patterns of endocytosis and lysosomal related genes in corals associated with clade D on 10days.

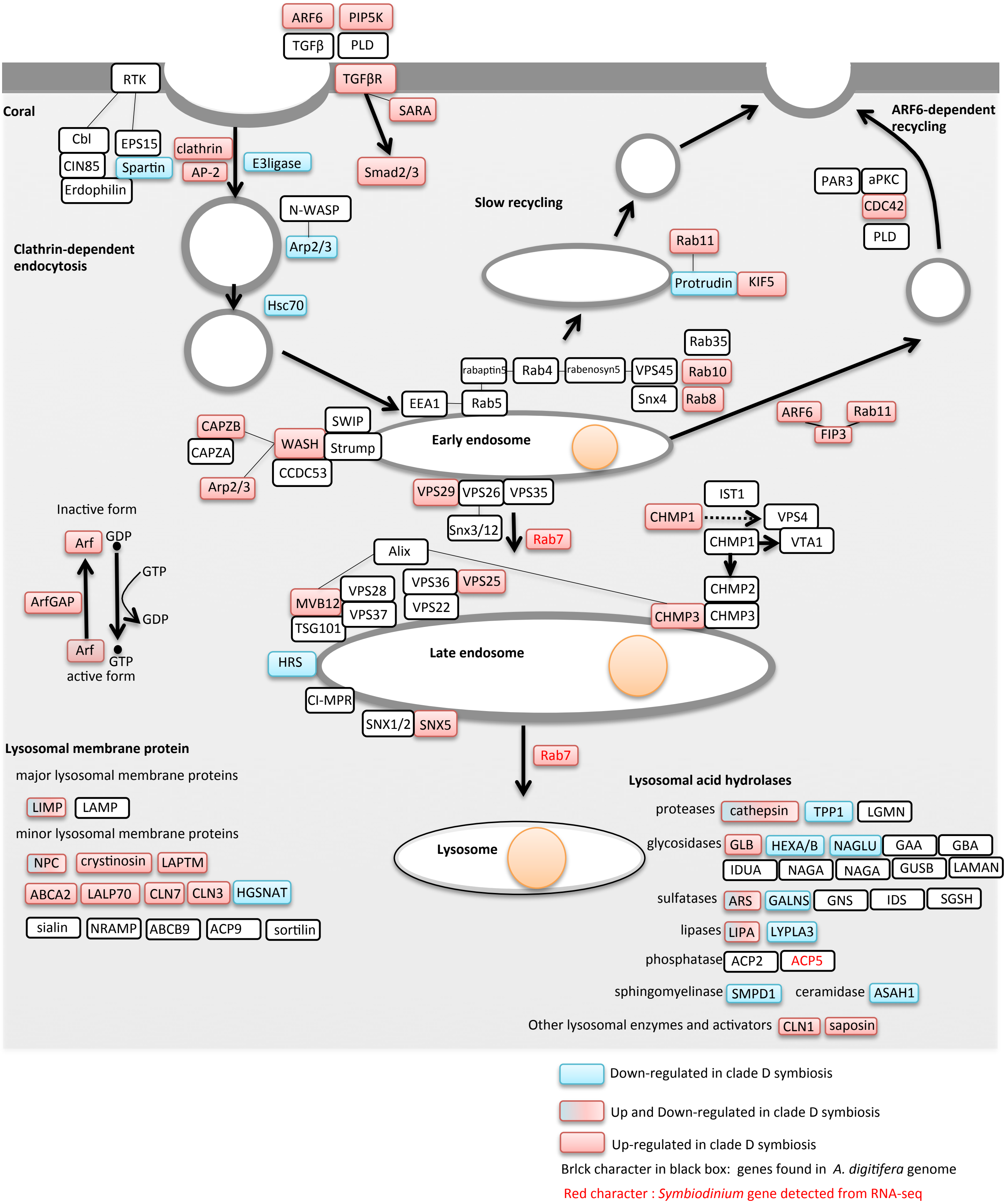

Fig. S5c. Gene expression patterns of endocytosis and lysosomal related genes in corals associated with clade D on 20days.

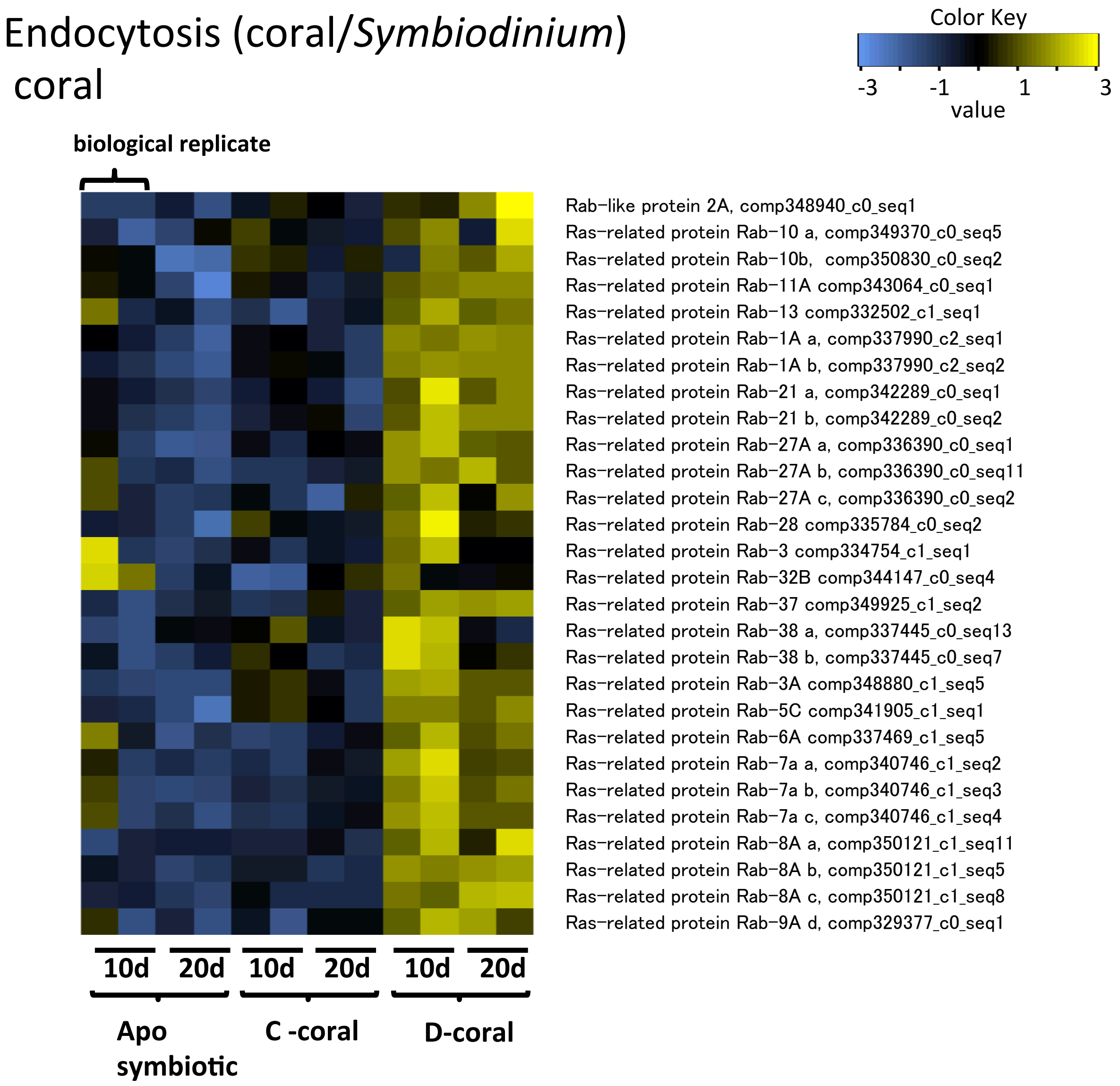

Fig. S6a. Heat map of the expression patterns of genes related to endocytosis.
Heat map shows the expression patterns of genes related to endocytosis in aposymbiotic corals, corals associated with clade C (C-coral) or clade D (D-corals) in 10 days and 20 days after inoculation of each *Symbiodinium*. Gnes whose expression were down-regulated were shown. There are selected notable treatment-specific genes (FDR P < 0.05).

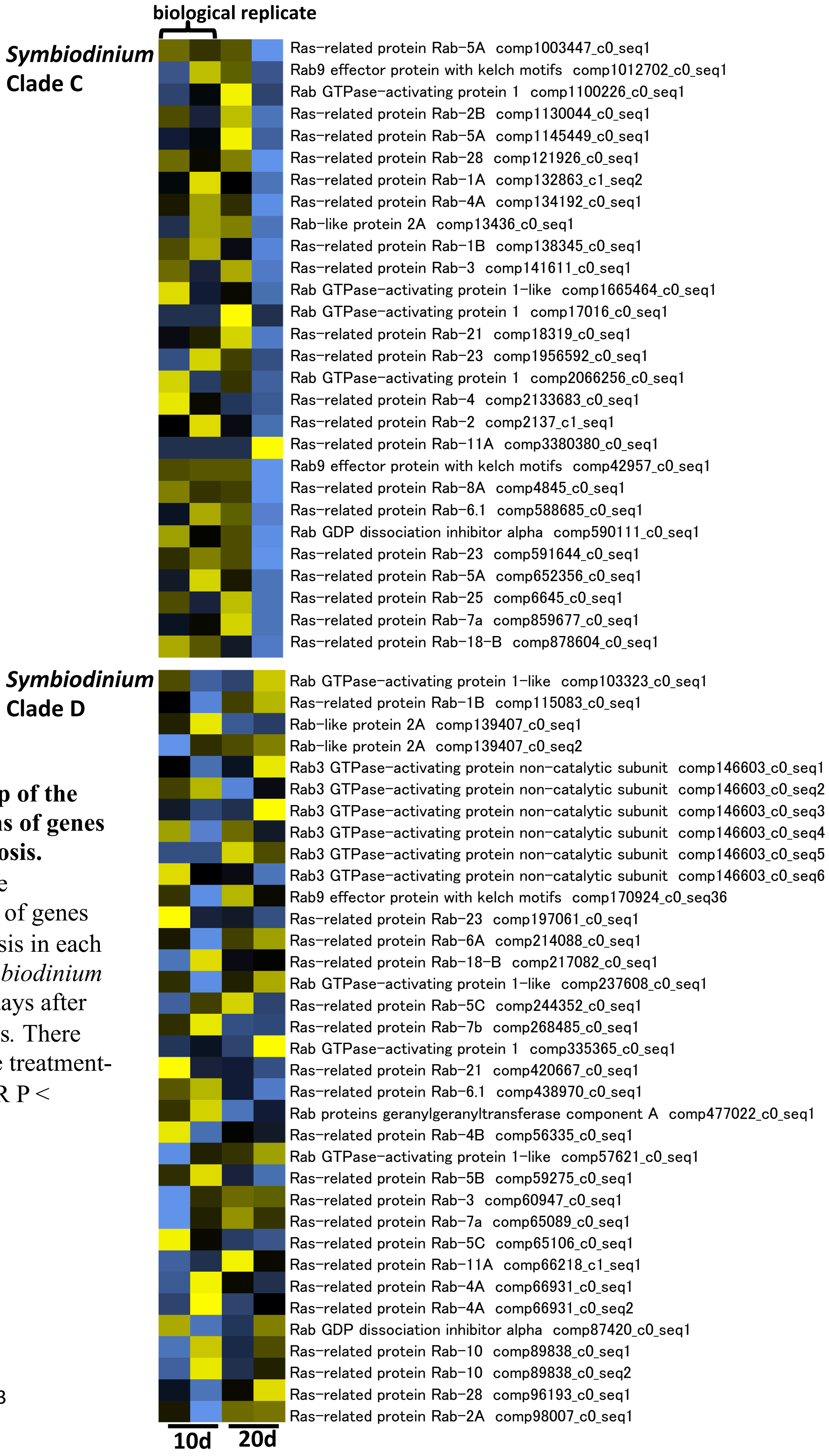

Fig. S6b. Heat map of the expression patterns of genes related to endocytosis. Heat map shows the expression patterns of genes related to endocytosis in each endosymbiotic *Symbiodinium* in 10 days and 20 days after inoculation to corals. There are selected notable treatment-specific genes (FDR P < 0.05).

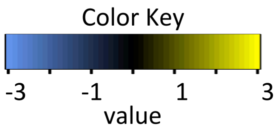

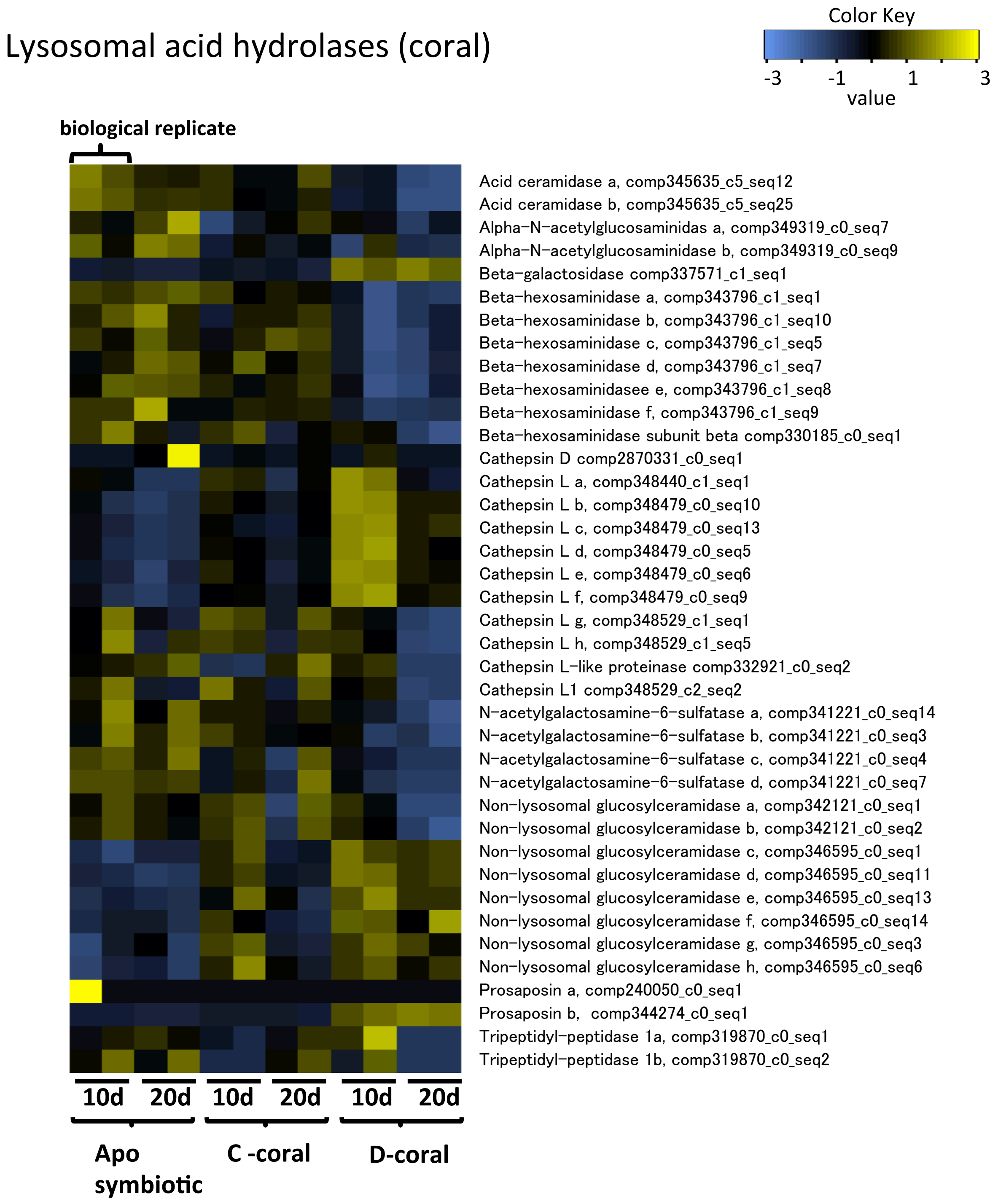

Fig. S7. Heat map of the expression patterns of genes related to lysosomal acid hydrolases. Heat map shows the expression patterns of genes related to lysosomal acid hydrolases in aposymbiotic corals, corals associated with clade C (C-coral) or clade D (D-corals) in 10 days and 20 days after inoculation of each *Symbiodinium.* Genes whose expression were down-regulated were shown. There are selected notable treatment-specific genes (FDR P < 0.05).

RNAi (*Symbiodinium*)

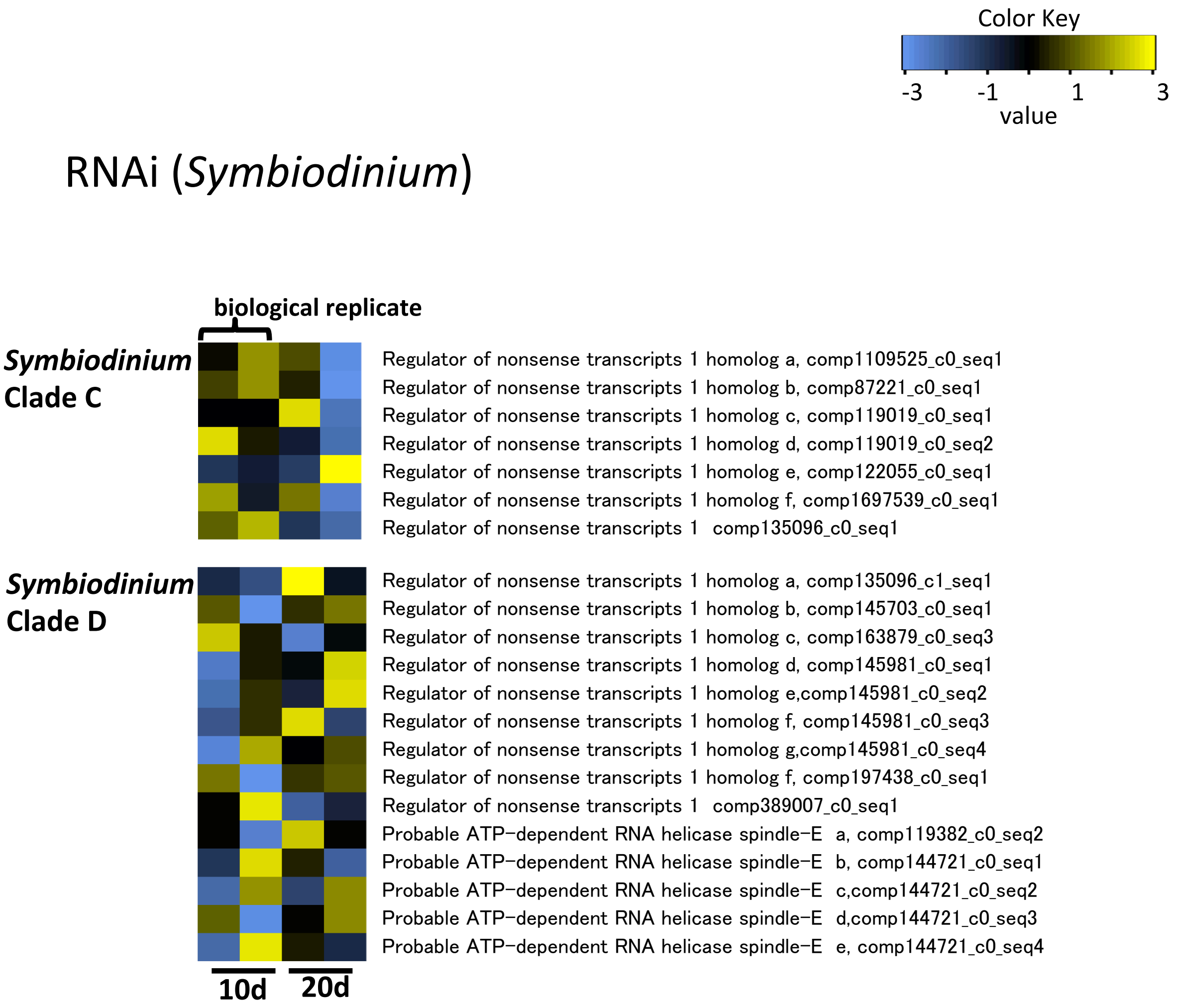

Fig. S8. Heat map of the expression patterns of genes related to RNAi.
Heat map shows the expression patterns of genes related to RNAi in each endosymbiotic *Symbiodinium* in 10 days and 20 days after inoculation to corals. There are selected notable treatment-specific genes (FDR P < 0.05).

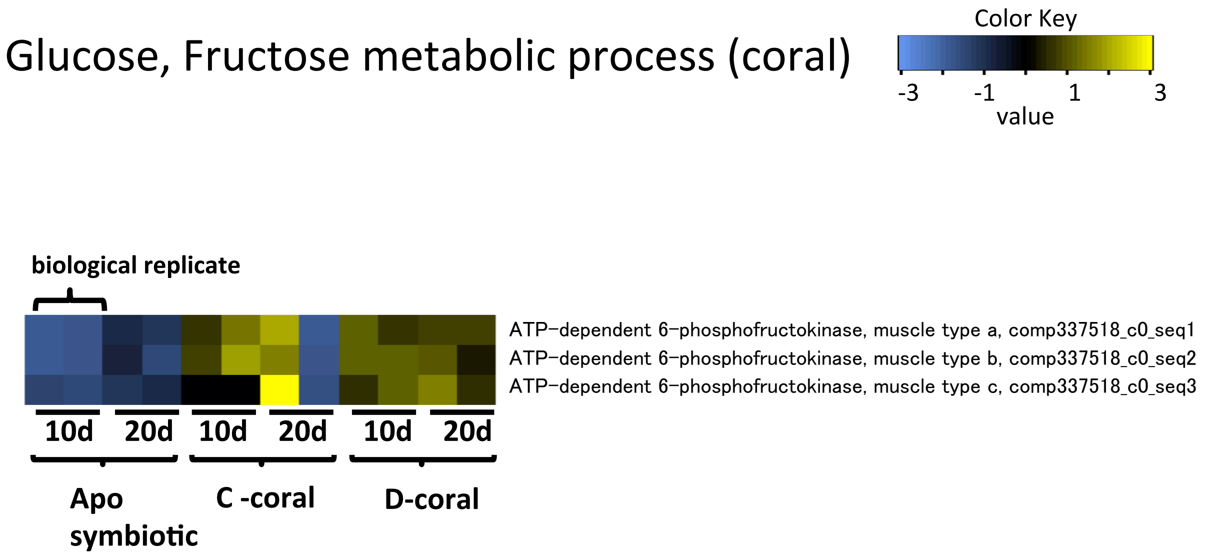

Fig. S9. Heat map of the expression patterns genes related to Glucose, Fluctose metabolic process. Heat map shows the expression patterns genes in in aposymbiotic corals, corals associated with clade C (C-coral) or clade D (D-corals) in 10 days and 20 days after inoculation of each *Symbiodinium*. There are selected notable treatment-specific genes (FDR P < 0.05).

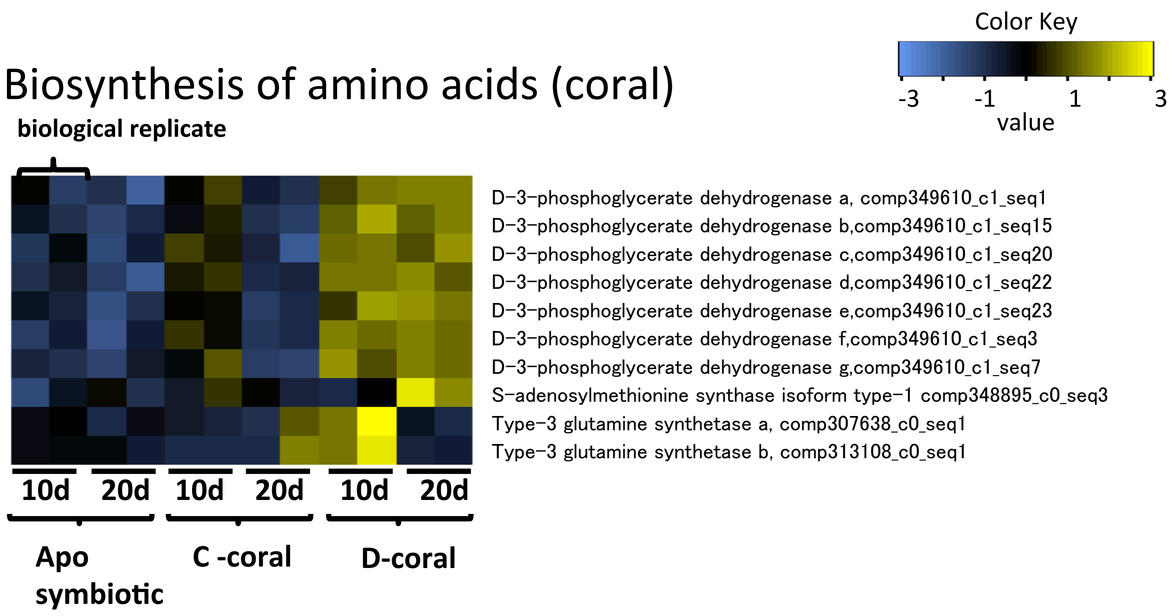

Fig. S10. Heat map of the expression patterns genes related to biosynthesis of amino acids. Heat map shows the expression patterns genes related to biosynthesis of amino acids in aposymbiotic corals, corals associated with clade C (C-coral) or clade D (D-corals) in 10 days and 20 days after inoculation of each *Symbiodinium*. There are selected notable treatment-specific genes (FDR P < 0.05).

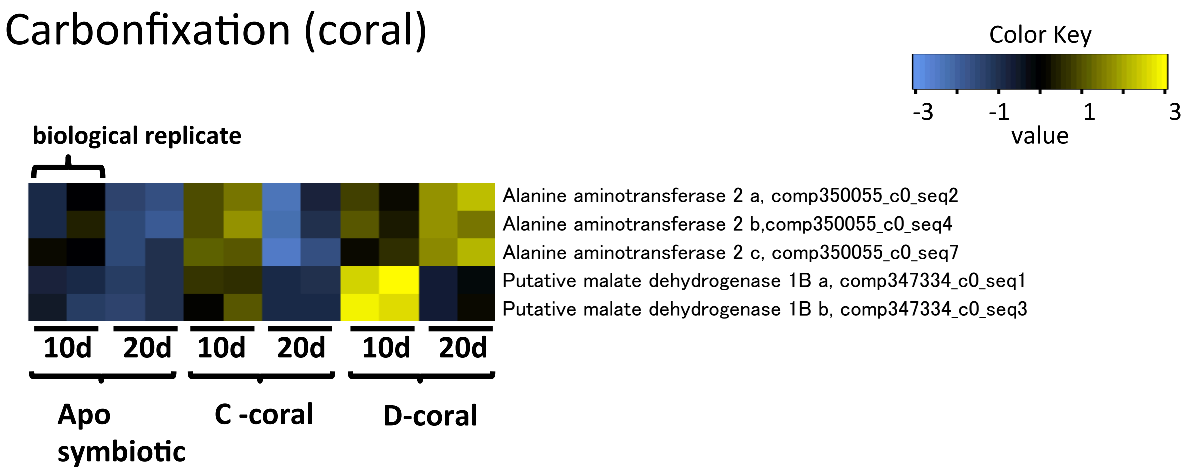

Fig. S11.Heat map of the expression patterns genes related to carbon fixation.
Heat map shows the expression patterns genes related to carbon fixation in aposymbiotic corals, corals associated with clade C (C-coral) or clade D (D-corals) in 10 days and 20 days after inoculation of each *Symbiodinium*. There are selected notable treatment-specific genes (FDR P < 0.05).

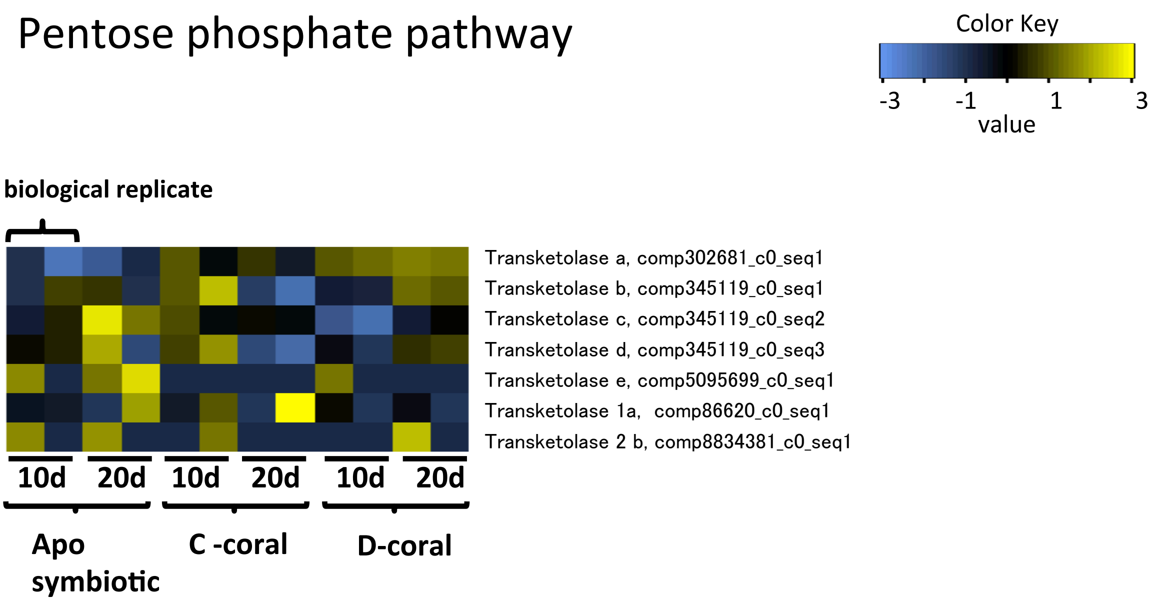

Fig. S12. Heat map of the expression patterns genes related to Pentose phosphate pathway. Heat map shows the expression patterns genes related to pentose phosphate pathway in aposymbiotic corals, corals associated with clade C (C-coral) or clade D (D-corals) in 10 days and 20 days after inoculation of each *Symbiodinium*. There are selected notable treatment-specific genes (FDR P < 0.05).

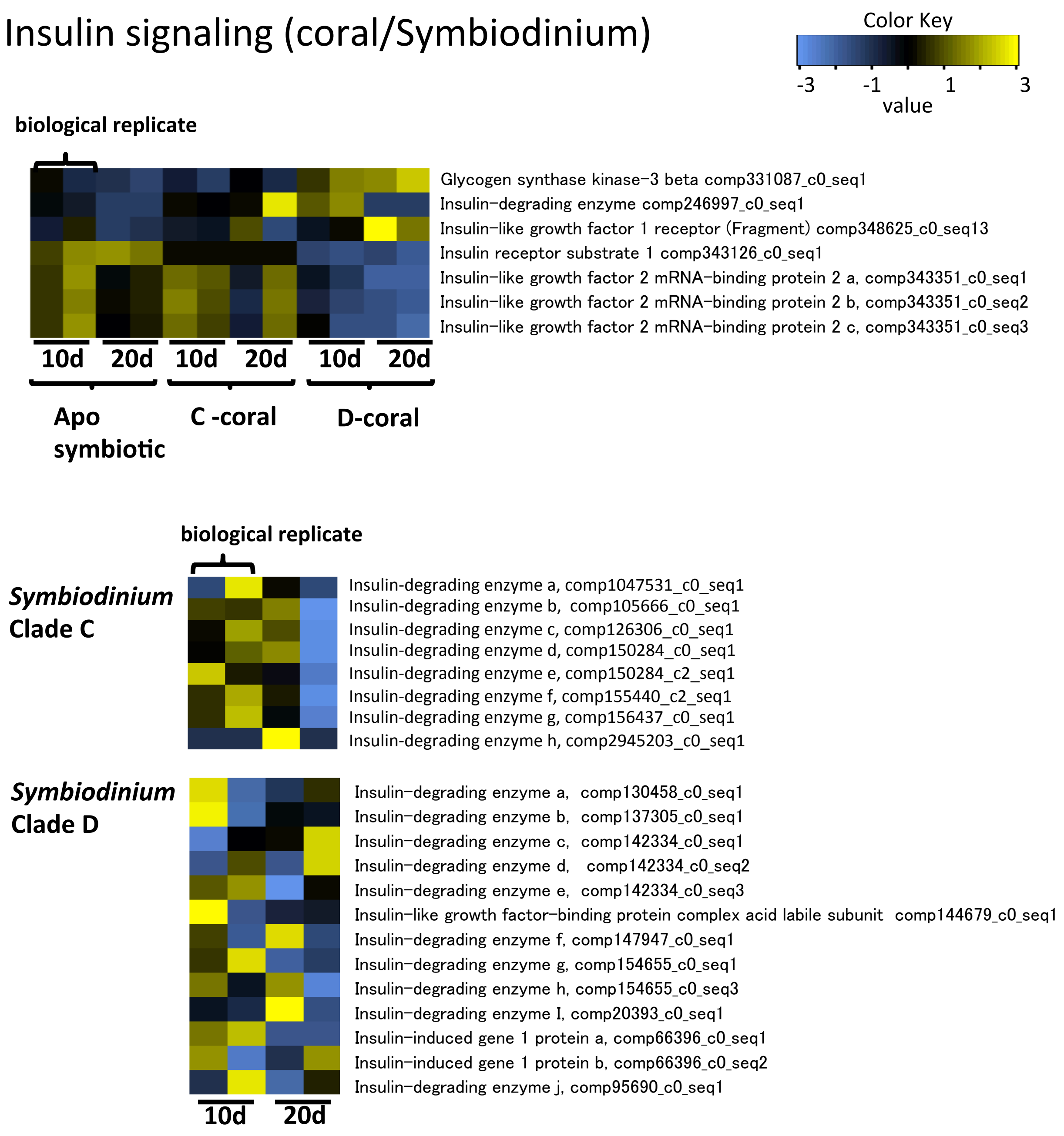

Fig. S13. Heat map of the expression patterns genes related to Insulin signaling.
Insulin signaling in aposymbiotic corals, corals associated with clade C (C-coral) or clade D (D-corals) in 10 days and 20 days after inoculation of each *Symbiodinium*. And expression data of Insulin-related genes of *Symbiodinium* are also shown. There are selected notable treatment-specific genes (FDR P < 0.05).

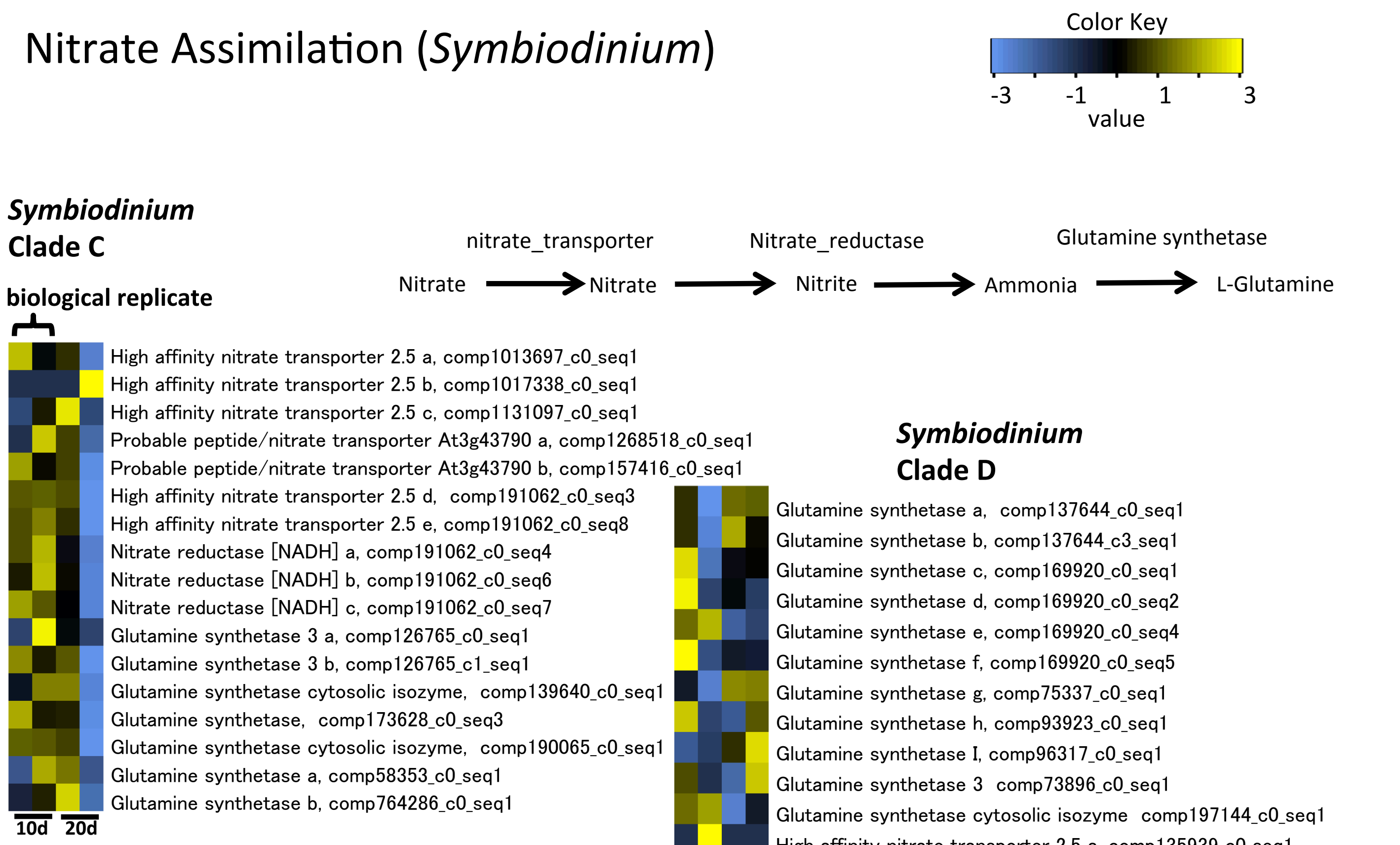

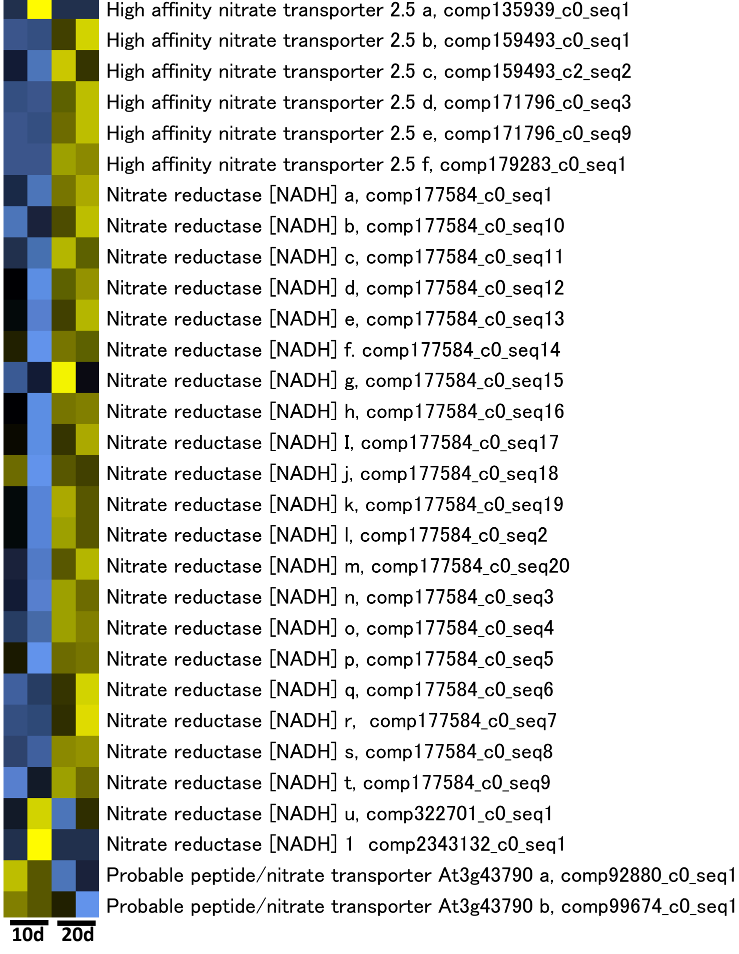

Fig. S14. Heat map of the expression patterns of genes involved in nitrate assimilation.
Heat map shows the expression patterns genes related to nitrate assimilation of *Symbiodinium*. There are selected notable treatment-specific genes (FDR P < 0.05).

Glutamate biosynthetic process (coral)

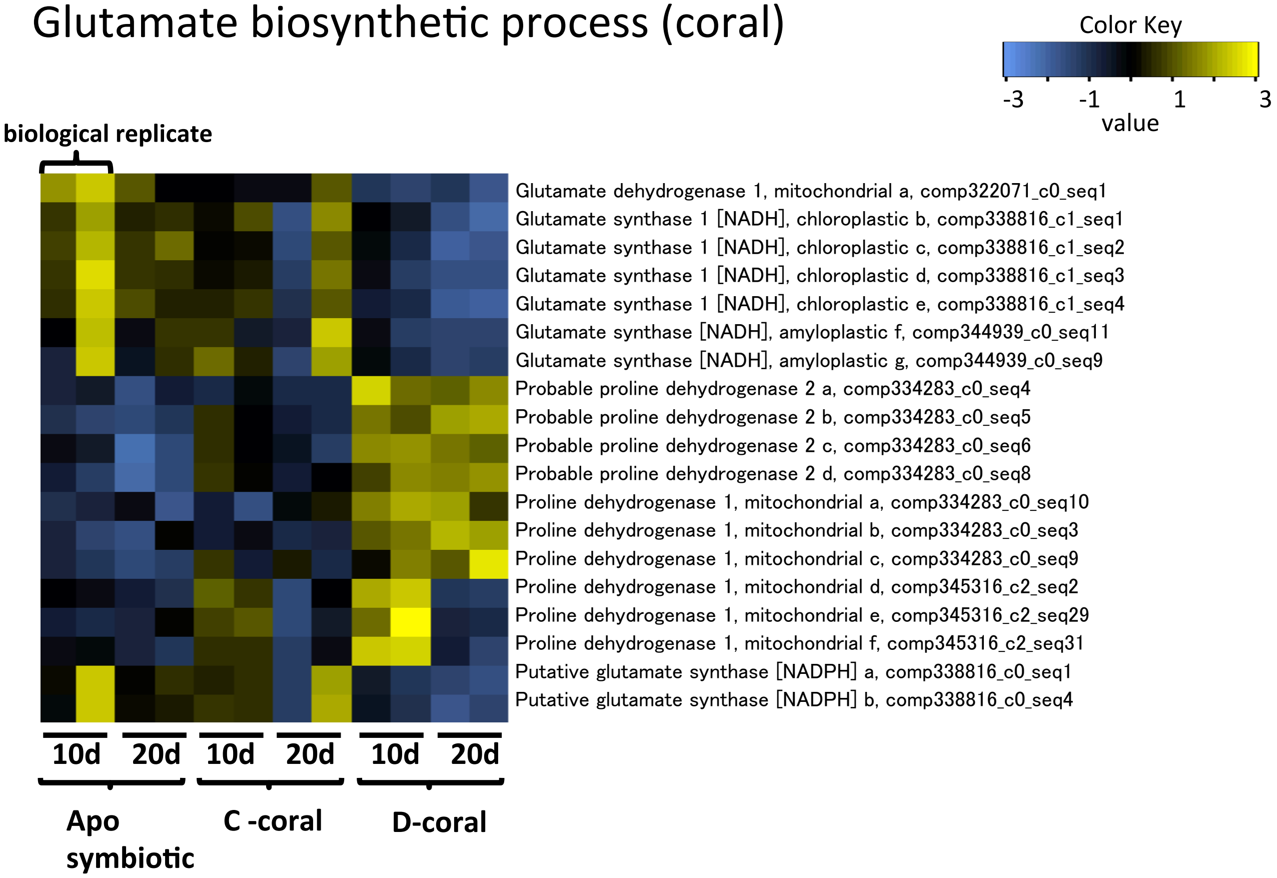

Osmoregulated proline transporter (*Symbiodinium*)

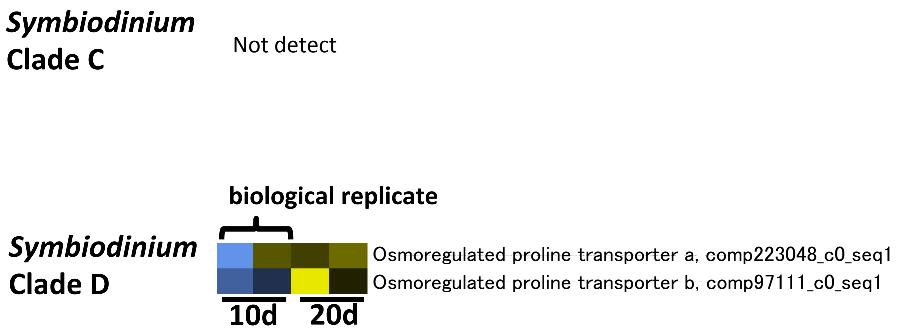

Fig. S15. Heat map of the expression patterns of genes related to glutamate biosynthetic process.

Heat map shows the expression patterns of genes related to glutamate biosynthetic process in aposymbiotic corals, corals associated with clade C (C-coral) or clade D (D-corals) in 10 days and 20 days after inoculation of each *Symbiodinium*. And expression data of genes coding Osmoregulated proline transporter of *Symbiodinium* are also shown. There are selected notable treatment-specific genes (FDR P < 0.05).

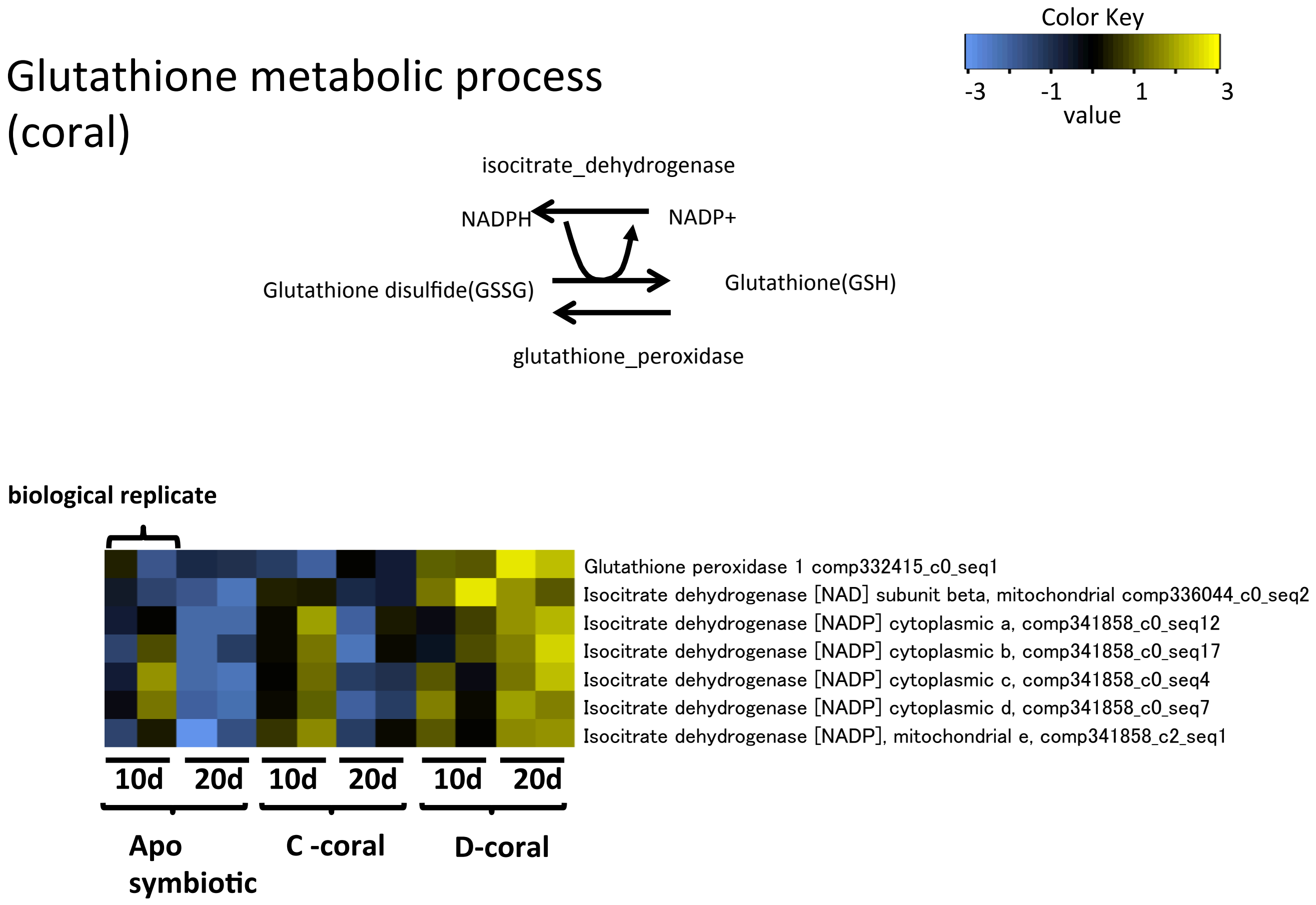

Fig. S16. Heat map of the expression patterns of genes related to glutathione metabolic process.

Heat map shows the expression patterns of genes related to glutathione metabolic process in aposymbiotic corals, corals associated with clade C (C-coral) or clade D (D-corals) in 10 days and 20 days after inoculation of each *Symbiodinium*. There are selected notable treatment-specific genes (FDR P < 0.05).

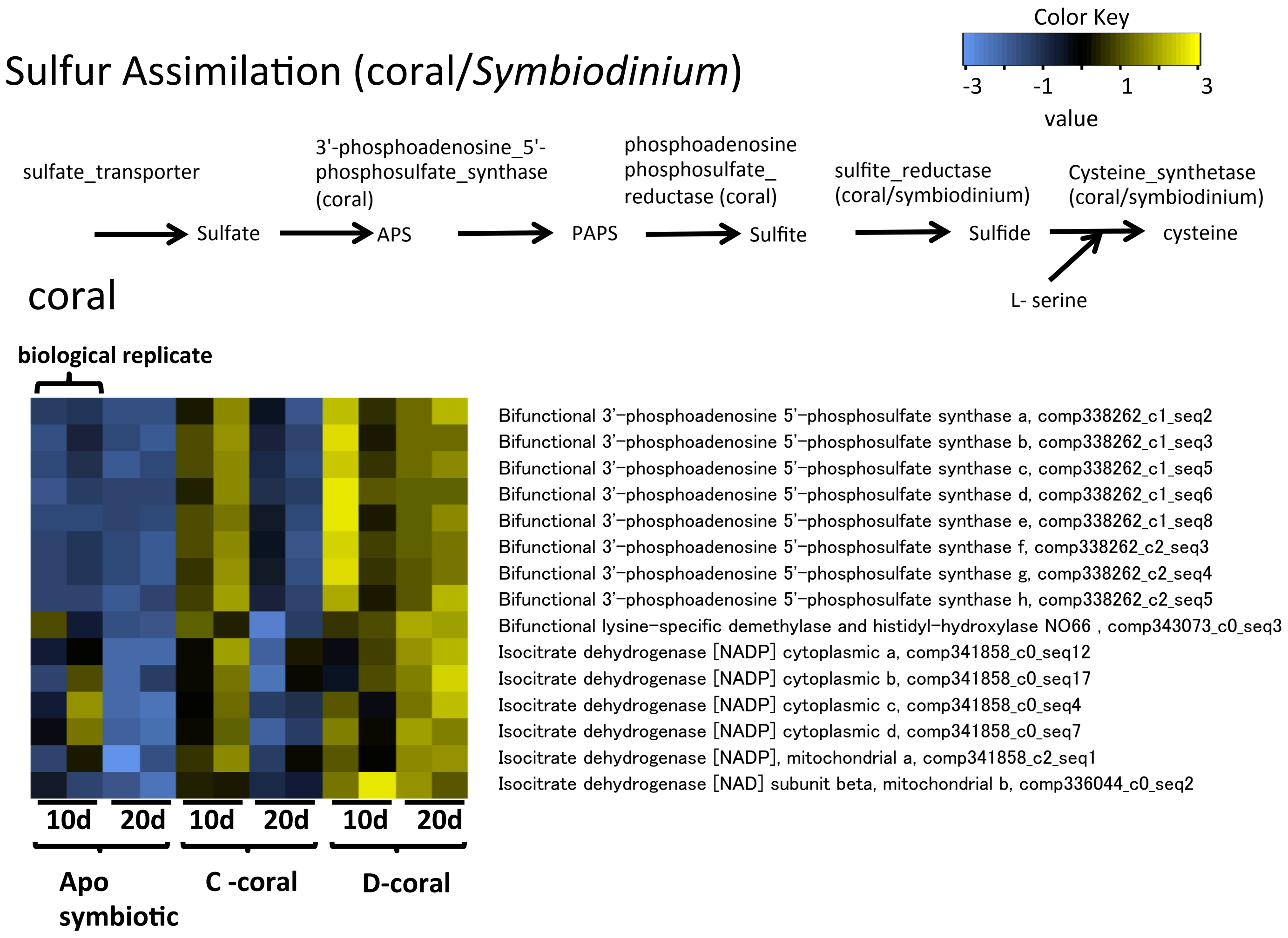

Fig. S17a. Heat map of the expression patterns of genes related sulfur assimilation. Heat map shows the the expression patterns of genes related sulfur assimilation in aposymbiotic corals, corals associated with clade C (C-coral) or clade D (D-corals) in 10 days and 20 days after inoculation of each *Symbiodinium.* There are selected notable treatment-specific genes (FDR P < 0.05).

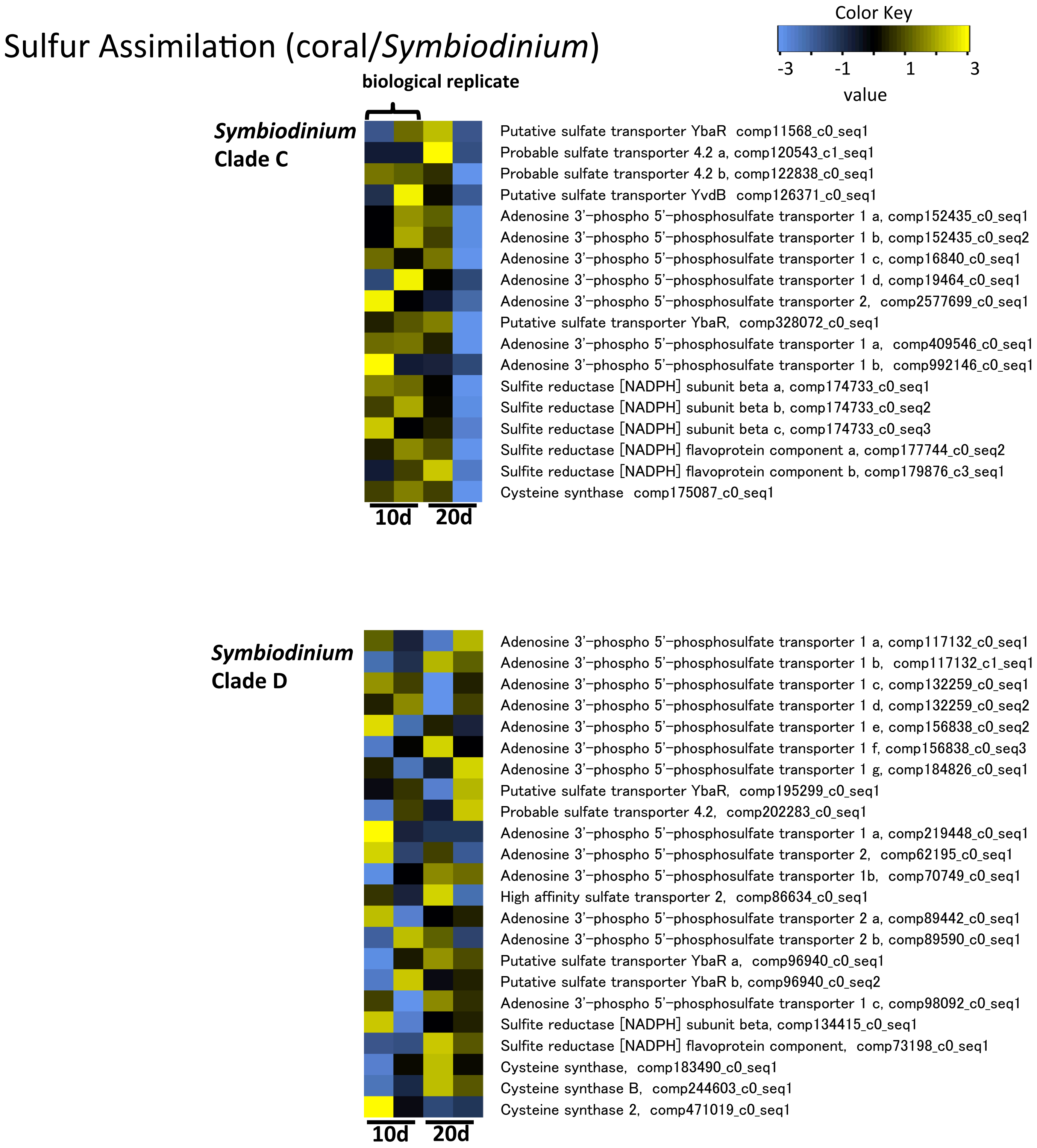

Fig. S17b. Heat map of the expression patterns of genes related to endocytosis.

Heat map shows the expression patterns of genes related to sulfur assimilation in each endosymbiotic *Symbiodinium* in 10 days and 20 days after inoculation to corals. There are selected notable treatment-specific genes (FDR P < 0.05).

Cyanoamino acid metabolism (coral)

coral

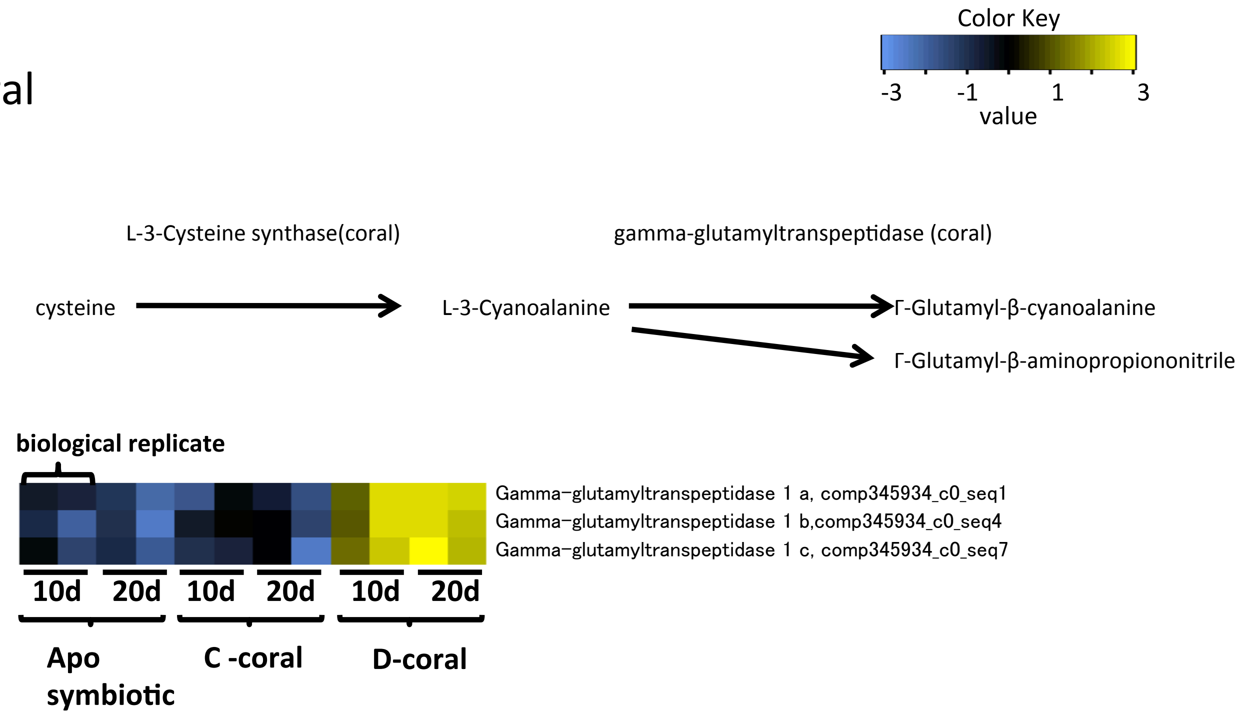

Fig. S18. Heat map of the expression patterns of genes related to cyanoamino acid metabolism.
Heat map show of the expression patterns of genes related to cyanoamino acid metabolism in aposymbiotic corals, corals associated with clade C (C-coral) or clade D (D-corals) in 10 days and 20 days after inoculation of each *Symbiodinium*. There are selected notable treatment-specific genes (FDR P < 0.05).

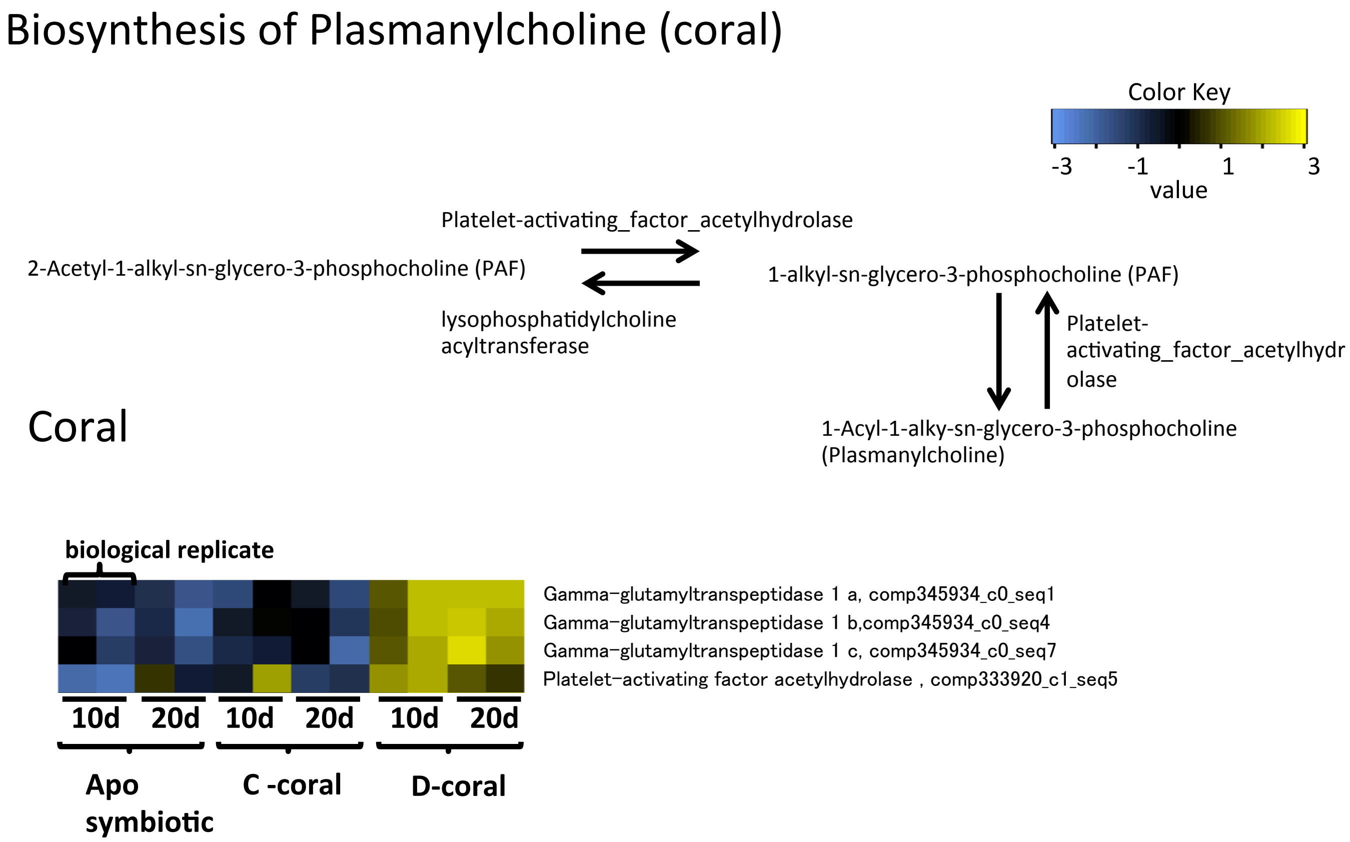

Fig. S19. Heat map of the expression patterns of genes related to biosynthesis of plasmanylcholine.
Heat map shows the expression patterns of genes related to biosynthesis of plasmanylcholine in aposymbiotic corals, corals associated with clade C (C-coral) or clade D (D-corals) in 10 days and 20 days after inoculation of each *Symbiodinium.* There are selected notable treatment-specific genes (FDR P < 0.05).

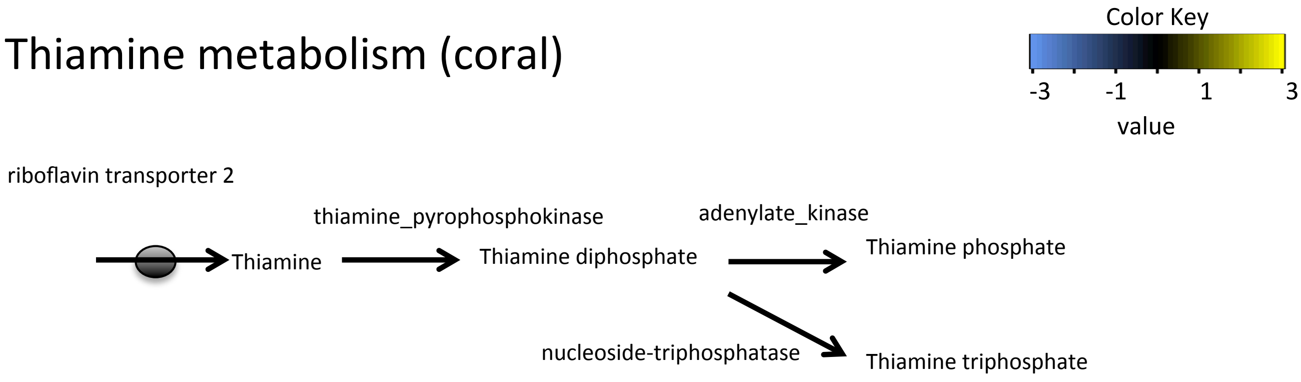

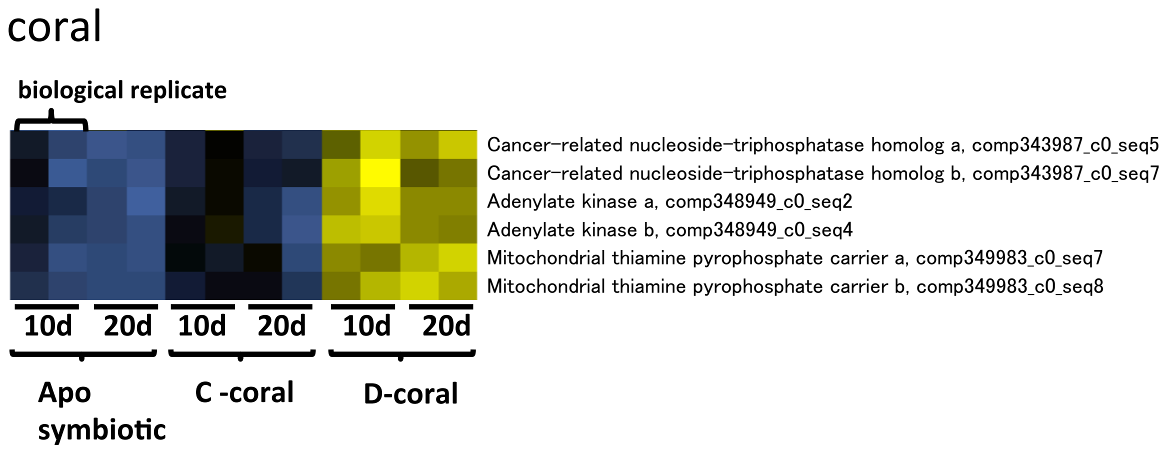

Fig. S20. Heat map of the expression patterns of genes related to thiamine (Vitamin B) metabolism.

Heat map shows the expression patterns of genes related to thiamine (Vitamin B) metabolism in aposymbiotic corals, corals associated with clade C (C-coral) or clade D (D-corals) in 10 days and 20 days after inoculation of each *Symbiodinium.* There are selected notable treatment-specific genes (FDR P < 0.05).

Gene expression pattern from the result of RNA-seq					The result of blastx			
gene_id	log2FoldChange in clade C-symbiosis on 10day	log2FoldChange in clade C-symbiosis on 20day	log2FoldChange in clade D-symbiosis on 10day	log2FoldChange in clade D-symbiosis on 20day	UniPlot KB accession	Protein name	Organism Name	E-value
comp343541_c0_seq2	-1.539859679	-1.957416973	-2.01262258	-4.10760851	Q9U6Y6	GFP-like fluorescent chromoprotein amFP486	Anemonia manjano	7.00E-112
comp343541_c0_seq6	-1.304746951	-1.870523076	-1.396355143	-3.306085	Q9U6Y6	GFP-like fluorescent chromoprotein amFP486	Anemonia manjano	2.00E-96
comp343541_c0_seq7	-1.60733518	-1.877147263	-2.048693528	-4.161228583	Q9U6Y6	GFP-like fluorescent chromoprotein amFP486	Anemonia manjano	7.00E-112
comp343546_c2_seq4	-1.065026917	-1.599162044	-1.415382344	-3.908150632				

Table. S1. Common differentially expressed genes (DEGs) in D-coral and C-coral on day10, day20.
